# Polarized Secretion of *Drosophila* EGFR Ligand from Photoreceptor Neurons Is Controlled by ER Localization of the Ligand-Processing Machinery

**DOI:** 10.1371/journal.pbio.1000505

**Published:** 2010-10-05

**Authors:** Shaul Yogev, Eyal D. Schejter, Ben-Zion Shilo

**Affiliations:** Department of Molecular Genetics, Weizmann Institute of Science, Rehovot, Israel; University of Zurich, Switzerland

## Abstract

The release of signaling molecules from neurons must be regulated, to accommodate their highly polarized structure. In the developing *Drosophila* visual system, photoreceptor neurons secrete the epidermal growth factor receptor ligand Spitz (Spi) from their cell bodies, as well as from their axonal termini. Here we show that subcellular localization of Rhomboid proteases, which process Spi, determines the site of Spi release from neurons. Endoplasmic reticulum (ER) localization of Rhomboid 3 is essential for its ability to promote Spi secretion from axons, but not from cell bodies. We demonstrate that the ER extends throughout photoreceptor axons, and show that this feature facilitates the trafficking of the Spi precursor, the ligand chaperone Star, and Rhomboid 3 to axonal termini. Following this trafficking step, secretion from the axons is regulated in a manner similar to secretion from cell bodies. These findings uncover a role for the ER in trafficking proteins from the neuronal cell body to axon terminus.

## Abstract 2

Trafficking within the endoplasmic reticulum and specialized localization of the intra-membrane protease Rhomboid regulate EGF ligand-dependent signaling in *Drosophila* photoreceptor axon termini.

## Author Summary

Cells secrete signaling molecules that trigger a variety of responses in neighboring cells by activating their respective cell-surface receptors. Because many cells in an organism are polarized, regulating the precise location of ligand secretion is important for controlling the position and nature of the response. During the development of the compound eye of the fruit fly *Drosophila*, for example, a ligand of the epidermal growth factor family called Spitz (Spi) is secreted from both the apical and basal (axonal) poles of photoreceptor cells but with different outcomes. Photoreceptor cells are recruited to the developing eye following apical secretion of Spi. Conversely, basal secretion of this same ligand, at a significant distance from the cell body, triggers differentiation of cells in the outer layer of the brain. Although secretion of Spi is known to occur at both poles of the cell, one important question is how Spi and its processing machinery are trafficked throughout the length of the photoreceptor axon to achieve basal secretion. In this study we show that the key to axonal trafficking is the regulated localization of Spi and its processing machinery, including the intramembrane protease Rhomboid, to sites within the endoplasmic reticulum (ER), which extends along the length of the axon. Two different Rhomboid proteins are expressed in photoreceptor cells, but only one of them is localized to the ER. We show that this ER-localized Rhomboid is indeed necessary and sufficient for Spi processing at axon termini. Our work therefore demonstrates how variations in intracellular localization of conserved signaling components can alter signaling outcomes dramatically. It also highlights the importance of the ER in trafficking proteins along the axon.

## Introduction

Communication between cells and their environment entails the release and reception of signaling molecules. In polarized cells, such as epithelia or neurons, the unique cellular architecture imposes constraints on the precise sites where signal release and reception occur. For example, the distribution of axonal guidance receptors is restricted to specific proximal or distal axon segments [1]. Similarly, secretion of molecules from neurons must be highly polarized for the ligand to propagate in the appropriate receptive field. In some cases, ligand is secreted along the axon, where it interacts with ensheathing glia [2],[3], whereas in other cases ligand is secreted locally from cell bodies or growth cones [4]–[6]. Thus, polarized secretion is an essential aspect of ligand processing in neurons.

An example of ligand secretion from both cell bodies and axonal termini is that of the *Drosophila* epidermal growth factor receptor (EGFR) ligand Spitz (Spi). In the *Drosophila* eye imaginal disc, photoreceptors differentiate in the wake of a progressive morphogenetic furrow, which sweeps from the posterior of the disc to its anterior [7],[8]. Secretion of Hedgehog (Hh) from nascent photoreceptor cell bodies promotes the continued movement of the furrow [9],[10]. Photoreceptor neurons subsequently secrete the EGFR ligand Spi from their cell bodies, triggering neurogenesis in closely neighboring cells [11],[12].

Once specified as neurons, R1–R6 photoreceptor axons grow across the basal surface of the eye disc, funnel through the optic stalk, and reach the lamina, where they locally induce the differentiation of lamina cartridge neurons [13],[14]. Secretion of Hh from photoreceptor axon termini triggers an initial phase of neurogenesis in the lamina precursor cells, marked by the expression of Dachshund (Dac) and the EGFR itself [5]. The subsequent phase of lamina neurogenesis requires Spi, which is also locally delivered by the incoming retinal axons. EGFR activation by Spi in the lamina leads to the differentiation of five neurons in each cartridge, which express the pan neuronal marker ElaV [6]. Thus, local secretion of Spi at the two distinct poles of photoreceptor neurons controls neurogenesis in both the eye disc and the lamina. While the mechanisms that regulate Hh delivery to axons have been explored [4], how Spi is secreted from both cell bodies and axonal termini remains unknown.

Spi is the cardinal EGFR ligand throughout *Drosophila* development. It is broadly expressed as an inactive precursor [15]. Spi secretion is dependent on processing by the intramembrane protease Rhomboid-1 (Rho-1) [16]. The inactive Spi precursor is retained in the endoplasmic reticulum (ER) by a COPI-dependent mechanism [17]. Trafficking of Spi from the ER to the Rho-1 compartment requires the type II transmembrane protein Star (S) [18],[19]. Upon arrival at this late secretory compartment, Spi is cleaved by the Rho-1 protease and subsequently released to the extracellular milieu.

Rho-1 also cleaves the chaperone S, thereby rendering it incompetent to traffic additional Spi molecules [20]. We have previously shown that two additional Rhomboid family members, Rho-2 (also called Stet) and Rho-3 (also called Roughoid [Ru]), which are dedicated to oogenesis and eye development, respectively [21],[22], localize to the ER, as well as to the late secretory compartment [23]. Although Rho-2 and Rho-3, like Rho-1, promote Spi release from the late compartment, their ER presence attenuates EGFR signaling, primarily because of premature cleavage of S [23]. Thus, in photoreceptor neurons, Spi secretion from cell bodies is promoted by both Rho-1 and Rho-3 acting in the late compartment, with the ER activity of the latter also attenuating the overall levels of secreted ligand.

The presence of ER markers has been observed in axons and dendrites from various neurons [24],[25], and the ER has been suggested to be continuous in Purkinje cell axons [26]. However, the traditional role assigned to axonal ER is in localized translation of transported mRNA, rather than translocation of secreted proteins. Recently, a role for the ER in promoting trafficking of NMDA glutamate receptor to dendrites in cultured rat hippocampal neurons has been described [27].

Here we examined the mechanisms that regulate Spi release from axonal termini. We find that, unlike secretion from cell bodies, axonal secretion of Spi relies exclusively on Rho-3. Furthermore, the ability of Rho-3 to promote axonal secretion of Spi stems from its combined ER and late compartment localization. Supplementing an ER presence to Rho-1 or eliminating the ER localization of Rho-3 alternates their potencies vis-à-vis axonal Spi secretion. Our data indicate that the importance of the ER stems from its ability to promote axonal trafficking of Rhomboids, a feature that we suggest is linked to the extension of the ER throughout the axon. Finally, we characterize the apical compartment in which Spi is processed in cell bodies, and suggest that it is also present at axonal termini, where Spi is processed following trafficking along the axon. Our results show that subcellular localization of the EGFR-ligand-processing machinery in photoreceptors dictates the polarity of ligand secretion, and highlight the role of the ER in facilitating protein trafficking from the neuronal cell body to the axon terminus.

## Results

### EGFR Activation in the Lamina Cartridge Neurons Is Exclusively Mediated by Rho-3

**Figure 1:**
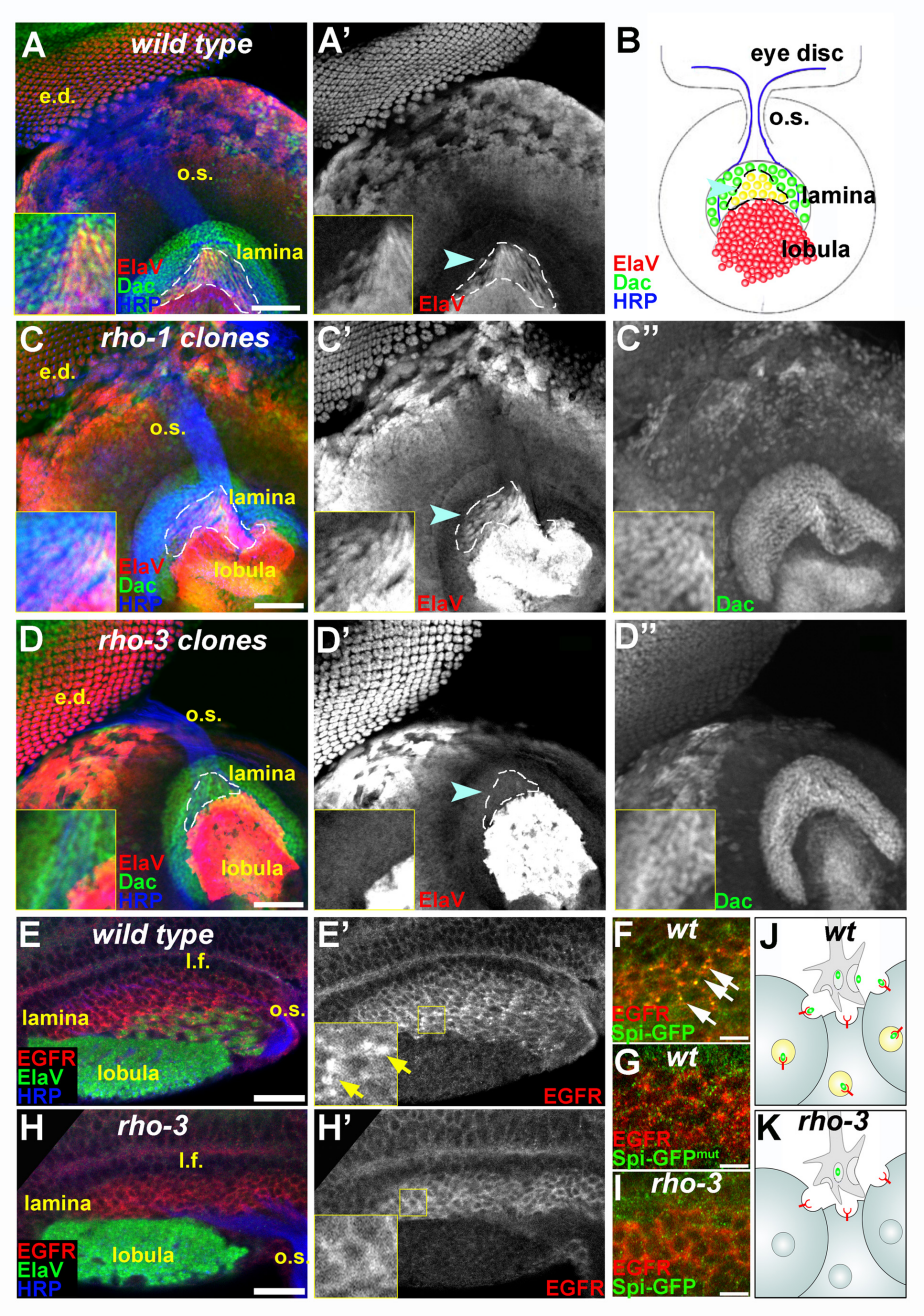
Rho-3 exclusively mediates Spi secretion from photoreceptor axons. (A–D) Lateral views of developing eye disc and lamina from late third-instar larvae. Photoreceptor cell bodies in the eye disc (e.d.) express the pan-neuronal marker ElaV (red, shown separately in single-primed panels). Photoreceptor axons, marked by HRP (blue), extend from the eye disc through the optic stalk (o.s.) and terminate at the lamina. The posterior lamina, in which ElaV is expressed, is marked by an arrowhead and outlined, and magnified in insets. Dac (green, shown separately in double-primed panels) and ElaV (red) expression in the lamina reflects Hh and Spi secretion from photoreceptor axons, respectively, and the triggering of the signaling pathways in the future lamina cartridge neurons. Scale bar: 40 µm. (A) In wild-type (wt) late third-instar larva, ElaV is expressed in the eye disc and lamina. (B) Schematic of (A). Note the retinotopic projections of photoreceptor axons in the lamina. At this developmental stage, not all photoreceptors have differentiated yet, hence only the posterior part of the lamina is invaded by retinal axons, and ElaV expression (yellow) is detected only there. (C) In eyes bearing large *rho-1* clones, ElaV and Dac are normally expressed in the lamina (inset), despite some morphological abnormalities. (D) Large *rho-3* clones eliminate EGFR activation in the lamina. ElaV expression is missing from the lamina (inset). Note that ElaV is still expressed in the eye disc, indicating that Rho-1 and Rho-3 redundantly mediate Spi secretion from cell bodies. Dac is normally expressed in the lamina, demonstrating that *rho-3* mutants do not suffer from general secretion defects. Anti-HRP staining (blue) shows that *rho-3* axons are correctly targeted to the lamina. (E) Anti-EGFR staining (red) in wild-type lamina shows many endocytic puncta (inset in E′, arrows) at the posterior of the lamina, associated with the ElaV-expressing cells (green). Scale bar: 20 µm. l.f., lamina furrow. (F) Spi–GFP (green) expressed in the eye by GMR–Gal4 is secreted from photoreceptor axons, and co-localizes with EGFR (red) in endocytic puncta (arrows) in lamina cells. Scale bar: 10 µm. (G) Spi–GFP (green) in which the Rhomboid cleavage site was mutated fails to localize with EGFR (red) in the lamina cells. Scale bar: 10 µm. (H) In a lamina from *rho-3* mutants, EGFR distribution (red) shows a reduced number of endocytic puncta (inset in H′), suggesting that the receptor is not engaged by the ligand on the surface of lamina cells. ElaV expression (green) is specifically missing from the lamina. Scale bar: 20 µm. (I) Spi–GFP (green), expressed in the eye of *rho-3* mutants is not secreted from the axons, and does not co-localize with EGFR in lamina cells. Scale bar: 10 µm. (J and K) Schemes of Spi secretion from axons. In wild-type larvae (J), Spi (green ovals) is secreted from axons and co-localizes with EGFR (red) in endocytic puncta in lamina cells. In the absence of cleavage by Rho-3 (K), Spi fails to be secreted from photoreceptor axons, and does not co-localize with EGFR in the lamina, which, in turn, is not internalized.

To investigate the requirement for Rho-mediated cleavage in promoting Spi release from photoreceptor axons, we assessed the effect of *rho-1* or *rho-3* mutations on lamina neurogenesis. In late third-instar larvae, EGFR activation by Spi delivered from photoreceptor axons leads to the expression of the pan-neuronal marker ElaV at the posterior part of the lamina (Figure 1A and 1B). Visual systems rendered homozygous for a null *rho-1* allele, using the Eyeless Gal4 UAS Flip (EGUF) system [28], occasionally show some morphological defects, but ElaV expression in the lamina is not perturbed (Figure 1C). Thus, *rho-1* is dispensable for Spi release from photoreceptor axons. We next examined ElaV expression in *rho-3* EGUF clones (Figure 1D) or in homozygous mutant animals (Figure 1H). While ElaV is properly expressed in the eye disc and brain lobula, we could not detect any ElaV expression in the lamina, indicating that *rho-3* is essential for EGFR activation in this tissue. Thus, whereas Rho-1 and Rho-3 can redundantly promote Spi release from cell bodies in the eye disc, only Rho-3 mediates EGFR activation in the lamina.

Since Rho-3 is also involved in photoreceptor neurogenesis, the lack of EGFR activation in the lamina of *rho-3* mutants may be a secondary effect of defective neuronal development or axonal mistargeting. However, *rho-3* mutant photoreceptors properly express the pan-neuronal marker ElaV, as well as markers of specific photoreceptor subtypes (Figure 1D′ and unpublished data; [23]), demonstrating that the general program of photoreceptor differentiation is not perturbed. The only defect we observed at the larval stage is an extra number of neurons, at the expense of non-neuronal cells [23]. Importantly, no overt axonal targeting defects were detected in the mutant, as seen with anti–horseradish peroxidase (HRP) staining (Figure 1D). Furthermore, the normal expression of the Hh target genes *dac* (Figure 1D′′) and *EGFR* (Figure 1E) in the brain reveals that there is no general secretion defect in *rho-3* mutants. It thus appears that the *rho-3* mutant phenotype reflects a specific defect in processing and secretion of Spi from axon termini.

To critically test the functionality of *rho-3* mutant photoreceptors, we performed electroretinogram (ERG) recordings on adult flies (Figure S1). Photoreceptor neurons from wild-type or *rho-3* eyes properly depolarize in response to light. However, “on/off transients,” which represent the activity of the post-synaptic lamina neurons [29], are absent in *rho-3* ERG recordings, thus reflecting the defects in lamina neurogenesis. Conversely, “on/off transients” are detected in *rho-1* EGUF clones. Hence, in the absence of Rho-3, Rho-1 facilitates all aspects of photoreceptor development, but not the induction of EGFR activation in the lamina.

### Release of Spi from Axon Termini Depends on Cleavage by Rho-3 in Photoreceptor Neurons

Rhomboids promote EGFR signaling by processing the ligand Spi in the signal-sending cell prior to its secretion [30],[31]. This suggests that the lack of EGFR activation in *rho-3* mutant laminae stems from a failure in cleavage and secretion of Spi from photoreceptors. To follow Spi processing and secretion, we monitored the localization of Spi–green fluorescent protein (GFP), a biologically active variant of the ligand, tagged by GFP at the extracellular domain [19]. The construct was expressed under the control of GMR–Gal4 [32], to restrict expression exclusively to the eye disc.

Inspection of EGFR distribution in the laminae of wild-type flies reveals many endocytic puncta, which are associated with the ElaV-expressing cartridge neurons (Figures 1E and S1D). We found that Spi–GFP secreted from the eye co-localized in the lamina with EGFR in these puncta, reflecting the release of the ligand from photoreceptor axons and endocytosis of ligand–receptor complexes by lamina cells (Figure 1F and 1J). This co-localization is dependent on cleavage by Rhomboid proteases, since a similarly expressed Spi–GFP construct in which the Rhomboid cleavage site was mutated [33] failed to co-localize with the receptor (Figure 1G and 1K).

We next examined the distribution of EGFR in *rho-3* mutant laminae, and found that it is uniform compared to wild-type, and lacks the bright endocytic puncta (Figures 1H and S1E). In *rho-1* mutant visual systems, the distribution and intensity of laminar EGFR staining were comparable to wild-type (Figure S1F). Furthermore, following expression of Spi–GFP in *rho-3* mutant eye discs, GFP-positive puncta could not be detected in the laminae (Figure 1I and 1K). These results indicate that Rho-3 cleaves Spi within the transmembrane domain in photoreceptor neurons, to promote ligand release from their axons to the lamina.

In summary, our results show that, whereas both Rho-1 and Rho-3 are capable of mediating Spi secretion from cell bodies in the eye disc, only Rho-3 promotes the secretion of Spi from photoreceptor axons to the lamina.

### Spi Secreted from R2 and R5 Photoreceptor Axons Patterns the Lamina

Each of the approximately 750 ommatidia in the *Drosophila* eye contains eight photoreceptor neurons of distinct identities. R1–R6 neurons project their axons to the lamina, whereas R7 and R8 project their axons to the medulla. To ask which of these neurons provides Spi for patterning the lamina, we used a repertoire of Gal4 lines to drive Rho-3 expression in different subsets of photoreceptors, and monitored their ability to rescue the *rho-3* mutant phenotype. All Gal4 drivers used are normally expressed in *rho-3* mutant eye discs (unpublished data). As a complementary assay, we expressed Spi–GFP with the same lines, and monitored its co-localization with the internalized EGFR in the signal-receiving lamina neurons. Our findings are summarized in Table 1, showing that Rho-3 acts to promote Spi secretion from the axons of R2 and R5. We note that these axons also play a pivotal role in axonal pathfinding, as their mistargeting can lead to defective guidance of the entire ommatidial fascicle [34]. The concordance between the assays of ElaV induction and Spi internalization in the lamina suggests that the difference between the photoreceptors that do or do not provide the signal lies in their ability to process or secrete Spi, rather than in the capacity of the lamina cells to respond only to Spi that is secreted from distinct photoreceptors.

**Table 1:** Spi is secreted to the lamina mainly from R2 and R5 photoreceptor axons.

Driver	Expressed in Photoreceptors	Rescue of *rho-3* Phenotype by UAS–Rho-3	Co-localization of Spi–GFP with EGFR in Lamina Cells
GMR–Gal4	R1–R8	+++	+++
MT14–Gal4	R2, R5, R8	+++	+++
Lz–Gal4	R1, R6, R7	+	−
K25–Gal4	R3, R4, R7	−	−
Mδ0.5–Gal4	R4, weak R3	−	−
Sca–Gal4	R8	−	−

Six Gal4 lines, expressed in different combinations of photoreceptor cells, were used to determine which neurons secrete Spi to the lamina. The ability of UAS–Rho-3 to rescue the *rho-3* phenotype and the co-localization of Spi–GFP with EGFR in lamina cells were assayed. Both experiments indicate that mainly R2 and R5 photoreceptor axons are responsible for delivering Spi to the lamina. –, no rescue (no laminar ElaV expression) or no co-localization; +++, full rescue, leading to wild-type ElaV expression, or co-localization of more than 90% Spi–GFP puncta with EGFR. Rescue with Lz–Gal4 (+) yielded ∼20% of the wild-type number of ElaV-expressing cells in the lamina.

### The Cytoplasmic Tail and First Intraluminal Loop Mediate Different Subcellular Localizations of Rho-1 and Rho-3

A mechanism that may account for the importance of Rho-3 in promoting Spi secretion from axons is RNA transport and localized translation. However, we have found no *rho-3* RNA in axons, even after Rho-3 overexpression, which rescues the *rho-3* phenotype (Figure S2). We have previously shown that Rho-1 and Rho-3 differ in their subcellular localization within photoreceptor cell bodies. When ectopically expressed with the Gal4–UAS system, Rho-1 localized to apical punctate structures, whereas Rho-3 was localized to the ER, as well as to the apical puncta [23]. We set out to test the hypothesis that the distinct intracellular localizations of Rho-1 and Rho-3 account for the difference in their capacity to trigger Spi processing and secretion in photoreceptor axons.

**Figure 2:**
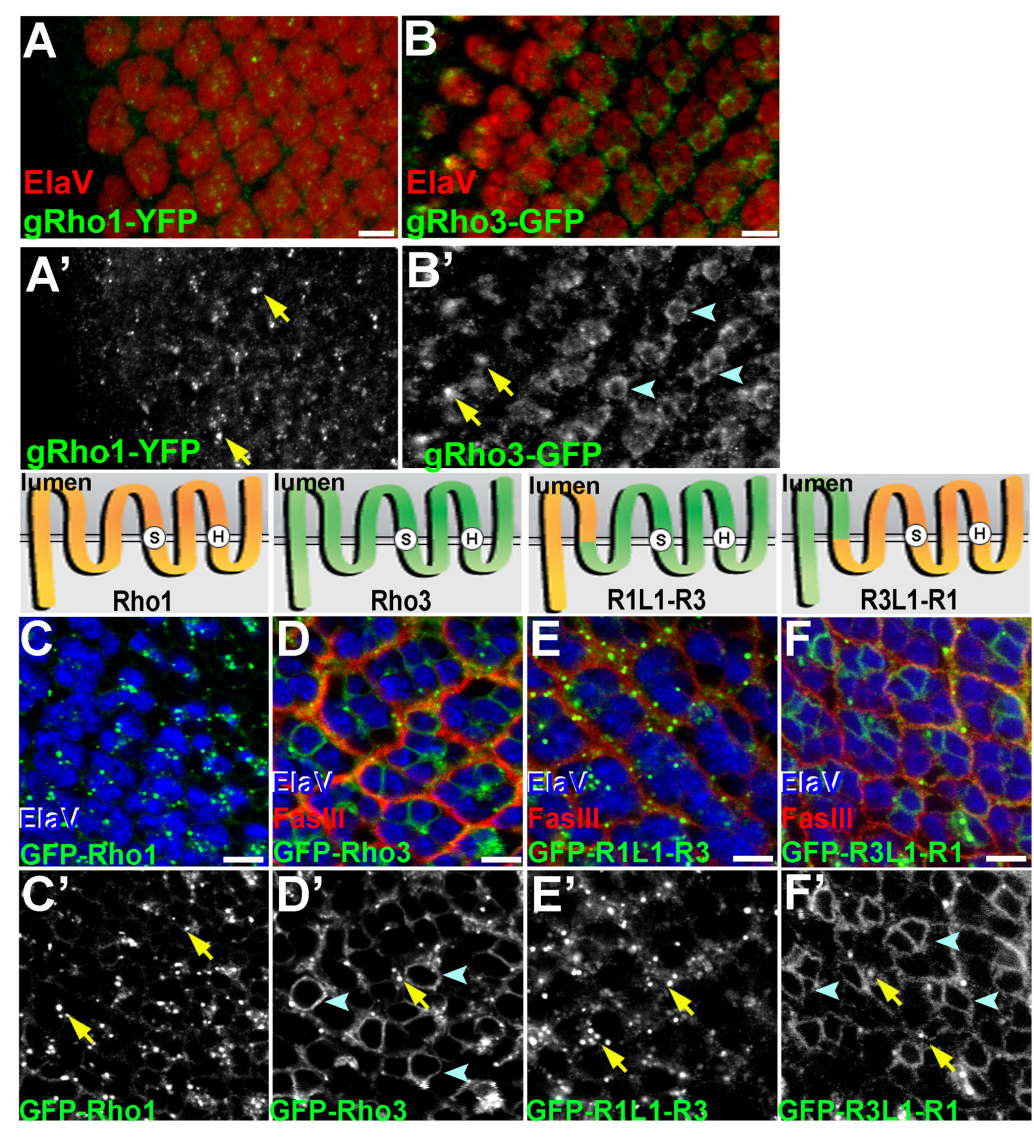
Subcellular localization of Rhomboids is mediated by their cytoplasmic N-termini and first luminal loop. (A) A gRho-1 construct, YFP tagged at the C-terminus (green), localizes to discrete punctate structures (arrows in [A′]). ElaV (red) shows photoreceptor nuclei. The morphogenetic furrow is to the left. Scale bar: 5 µm. (B) Rho-3, expressed from a genomic construct and tagged with GFP at the C-terminus (green) shows both ER (arrowheads in [B′]) and punctate (arrows in [B′]) localization. Like the Rho-1 puncta, Rho-3 puncta are more abundant in apical optical sections (not shown). ElaV (red) shows photoreceptor nuclei. Primed panels show single channels for YFP or GFP. Scale bar: 5 µm. (C–F) Subcellular localization of GFP-tagged Rho-1, Rho-3, R1L1-R3, and R3L1-R1 (green), expressed in the eye disc by GMR–Gal4. ElaV (blue) marks photoreceptor nuclei, and FasIII (red) stains membranes. Primed panels show a single channel for GFP. Scale bar: 5 µm. The schemes at the top of each panel show the topology of the proteases (N-termini are in the cytoplasm; C-termini are luminal; not to scale). Also shown are the positions of the catalytic serine (S) and histidine (H), embedded in the fourth and sixth transmembrane helices, respectively. Rho-1 is orange; Rho-3 is green. (C) GFP–Rho-1 localizes to apical punctate structures (arrows in [C′]). (D) GFP–Rho-3 localizes to the apical structures (arrows in [D′]) and the peri-nuclear ER (arrowheads in [D′]). (E) The N terminus and first luminal loop of Rho-3 were replaced with that of Rho-1. These sequences are sufficient to confer a Rho-1-like localization to GFP–R1L1-R3 (arrows in [E′]). (F) Rho-1 in which these sequences are derived from Rho-3 (GFP–R3L1-R1) is localized to the ER (arrowheads in [F′]) and the apical puncta (arrows in [F′]).

First, we examined the endogenous localization of the two proteases, without resorting to overexpression. Since antibodies that recognized the endogenous proteins could not be raised, we used recombineering [35] to generate ∼45-kb genomic fragments encompassing the *rho-1* or *rho-3* locus that express C-terminally tagged Rho-1–yellow fluorescent protein (YFP) and Rho-3–GFP in patterns and levels identical to the endogenous proteins. Transgenic lines were generated, in which the recombineered genes were inserted at the same chromosomal location. In the eye disc, genomic Rho-1 (gRho-1)–YFP localized exclusively to the apical compartment, whereas gRho-3–GFP was enriched in the ER, with staining also at the apical compartment (Figure 2A and 2B). These distributions demonstrate that despite the caveats associated with overexpression, the localizations obtained previously by the UAS–Gal4 system faithfully reflected the endogenous localization of these proteins.

To identify the sequences mediating the subcellular localization of Rhomboids, we swapped different fragments between Rho-1 and Rho-3. The resulting chimeras were GFP tagged, and transgenic animals were generated. In all cases the constructs were inserted at the same genomic location, to avoid a difference in expression levels. We find that the subcellular localization of Rhomboids depends on their cytoplasmic N terminus and the first intraluminal loop. Replacing these fragments of Rho-1 with the corresponding fragments from Rho-3, to yield GFP–R3L1-R1, relocalized Rho-1 to a Rho-3-like distribution, encompassing the ER and apical compartment (Figure 2C and 2F). Conversely, Rho-3 in which the N terminus and first loop were replaced by those of Rho-1 (GFP–R1L1-R3) retained localization to the apical compartment, but was absent from the ER (Figure 2D and 2E). Importantly, since the active site of the proteases is formed by residues embedded within the fourth and sixth transmembrane helices [36]–[38], the chimeras uncouple the subcellular localization signal from the catalytic activity. Therefore, the GFP–R1L1-R3 and GFP–R3L1-R1 constructs allow us to specifically define the role of subcellular localization in promoting Spi secretion from axonal termini.

### ER Localization of Rho-3 Facilitates Spi Secretion from Axons

Although both Rho-1 and Rho-3 promote Spi secretion from photoreceptor cell bodies, only Rho-3 facilitates Spi secretion from axons. To investigate whether this is due to its ER localization, we assayed the ability of GFP–Rho-1 or GFP–Rho-3 to rescue the *rho-3* lamina phenotype. In addition, we tested a modified Rho-1 targeted to the ER and late compartment (GFP–R3L1-R1) and an ER-excluded Rho-3 (GFP–R1L1-R3) using the same assay. All constructs were shown to be efficient in cleaving Spi in cell culture assays and in vivo (unpublished data). Furthermore, since Rho-1 and Rho-3 are normally expressed at low levels in the eye disc, we inserted all the transgenes into attP18, a genomic landing site that was reported to yield low expression levels [39], and expression was driven in R2, R5, and R8 by MT14–Gal4.

**Figure 3:**
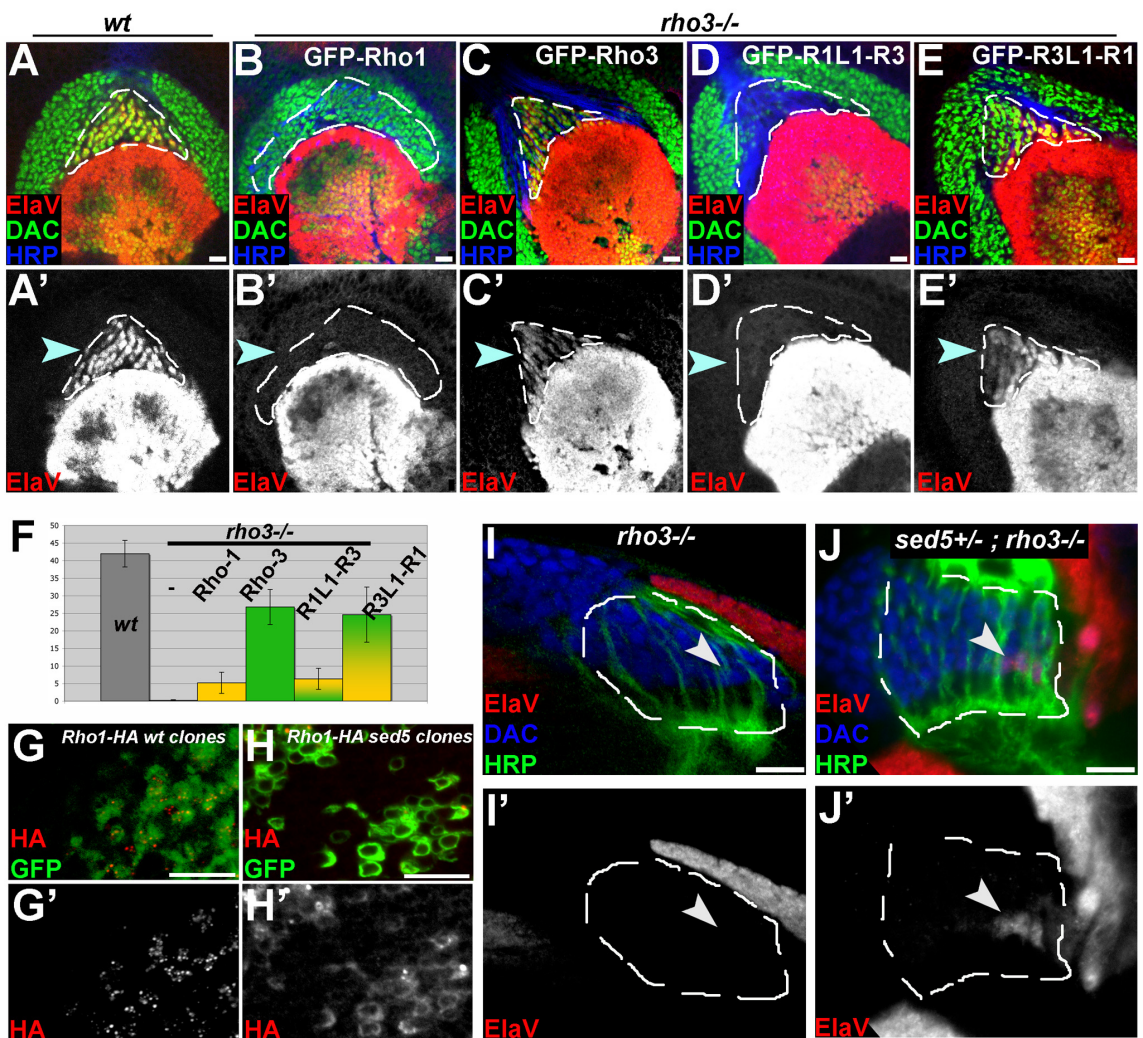
ER localization of Rho-3 facilitates Spi secretion from axons. (A–E) All constructs are GFP tagged at the N-termini, inserted into the same genomic location, and expressed in R2, R5, and R8 by MT14–Gal4. ElaV is red; Dac (green) and HRP (blue) mark photoreceptor axons. (A) Lateral view of a wild-type (wt) lamina from a late third-instar larva, with the typical ElaV triangle at the posterior. (B) GFP–Rho-1, expressed in R2, R5, and R8 fails to rescue the *rho-3* phenotype, as indicated by the lack of ElaV-positive cells within the population of Dac-positive precursors. (C) A GFP–Rho-3 transgene restores ElaV expression to the lamina of a *rho-3* mutant. (D) When Rho-3 is not localized to the ER, as is the case of the GFP–R1L1-R3 chimera, it fails to promote Spi secretion from the axons and induce EGFR activation in the lamina. (E) An ER-enriched Rho-1 (GFP–R3L1-R1) rescues the *rho-3* phenotype. (A′–E′) Single channel for ElaV staining. (F) Quantification of the results from (A–E). ElaV-positive cells in the lamina were counted in 8–10 specimens per genotype. The difference between ER-resident and non-ER-resident proteases is statistically significant (ANOVA). (G) Rho-1–HA (red, shown separately in [G′]) expressed in wild-type MARCM clones (marked by GFP, green) is localized to the typical apical puncta. (H) ER-to-Golgi trafficking is blocked in *sed5* MARCM clones, marked by GFP. Rho-1–HA (red, shown separately in [H′]) expressed in the mutant photoreceptors is retained in the peri-nuclear ER. (I) Horizontal view of a *rho-3* mutant lamina. HRP (green) marks retinal axons and outlines the lamina; Dac is blue. No ElaV-positive cells are seen in *rho-3* mutant lamina (red, shown separately in [I′]). (J) ElaV expression (red, shown separately in [J′]) is restored to a small population of cells at the posterior of the lamina of *rho-3* mutants after elimination of one copy of *sed5*. Scale bars: 10 µm.

As expected from their in vivo activities, GFP–Rho-3 rescued the *rho-3* mutant lamina phenotype, whereas GFP–Rho-1 did not (Figure 3). Importantly, while GFP–Rho-1 failed to promote Spi secretion from the axons, supplementing it with an ER localization yielded a construct (GFP–R3L1-R1) capable of rescuing the *rho-3* phenotype (Figure 1E and 1F). Conversely, whereas GFP–Rho-3 rescued the *rho-3* phenotype, a Rho-3 version which is not ER localized (GFP–R1L1-R3) failed to do so (Figure 1D and 1F). These experiments show that ER localization is a critical feature that enables Rhomboid proteases to promote Spi secretion from the axons.

We next asked whether intact endogenous Rho-1, which cannot substitute for Rho-3 in Spi processing for axonal release, can facilitate Spi secretion when enriched in the ER. Passage through the ER is an essential step in Rho-1 maturation, as a protein bearing transmembrane domains. We thus attempted to compromise Rho-1 exit from the ER, by removing one copy of the syntaxin *sed5*, which is required for the fusion of ER-derived vesicles with the Golgi [40],[41]. When HA-tagged Rho-1 was expressed in *sed5* homozygous mutant clones, its subcellular distribution shifted almost completely to the peri-nuclear ER (Figure 3G and 3H). In *rho-3* mutants in which *sed5* gene dosage was halved, we found that some ElaV expression was restored to the lamina (Figure 3I and 3J). Therefore, when endogenous Rho-1 trafficking out of the ER is compromised, it can substitute for Rho-3 and promote Spi release from axons.

We note here that under strong overexpression conditions, Rho-1 also rescues the *rho-3* phenotype. This may reflect the perdurance of some Rho-1 in the ER when its export machinery is heavily burdened. Indeed, a low endogenous level of ER activity by Rho-1 en route to the apical compartment has been suggested previously [17]. Accordingly, the ER levels of Rho-1–HA in *sed5* heterozygotes were too low to be detected by anti-HA staining, yet restored some laminar ElaV expression to *rho-3* mutants. In summary, our results indicate that the difference in subcellular localization is the cause of the distinct ability of Rho-3, but not Rho-1, to promote Spi processing and secretion from photoreceptor axons.

### Spi Processing for Axonal Signaling Does Not Take Place in the ER

**Figure 4:**
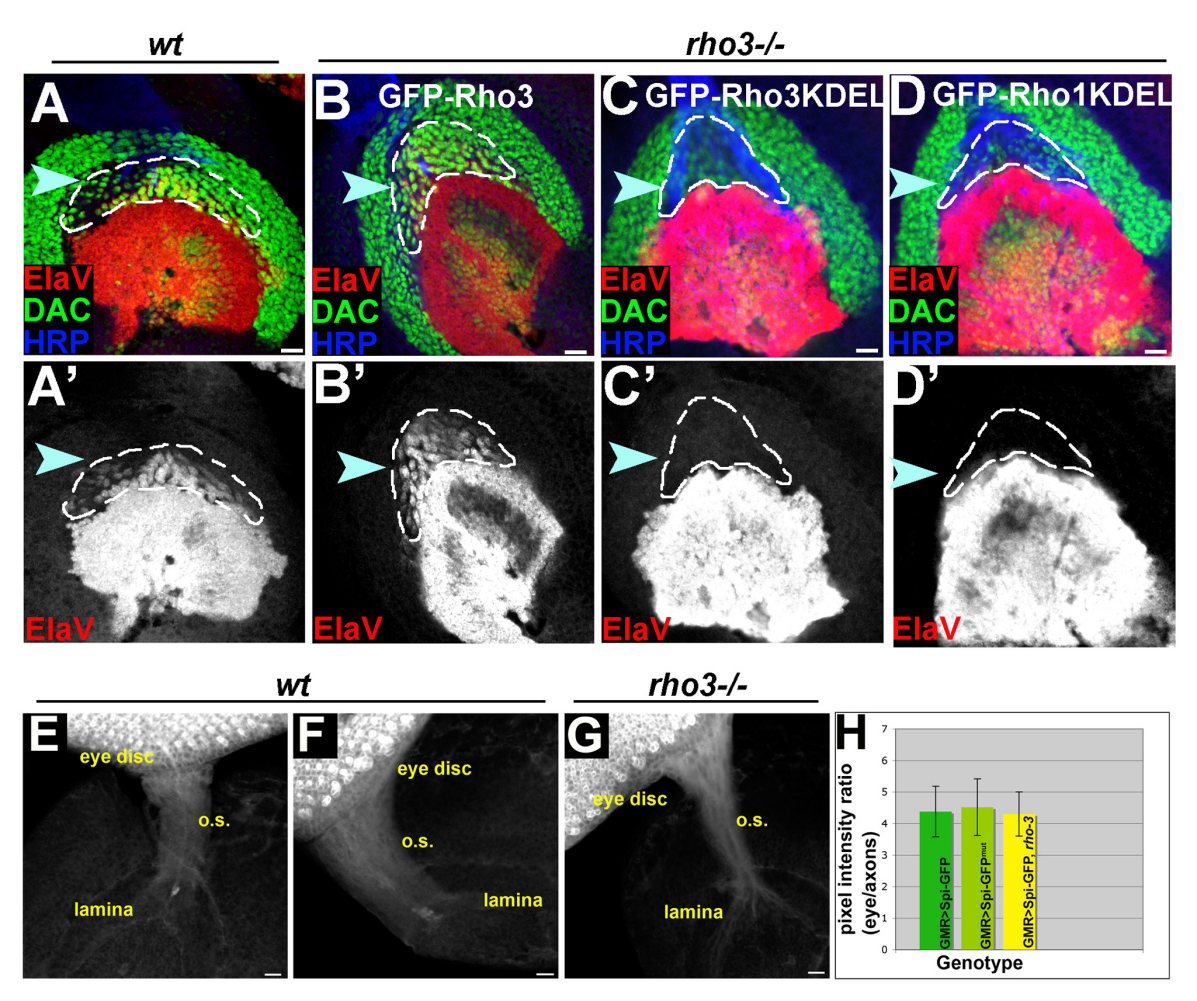
Spi secreted from axons is not processed in the ER. (A–D) Lateral views of late third-instar larva laminae. ElaV (red, shown separately in primed panels), Dac (green), and HRP (blue). The posterior of the lamina, where EGFR activation is evident by ElaV expression, is marked with an arrowhead and is outlined. (A) Wild-type (wt) lamina showing the typical ElaV staining at its posterior. (B) A GFP–Rho-3 transgene, expressed in R2, R5, and R8 by MT14–Gal4 restores ElaV expression in *rho-3* mutants (compare with Figure 3B). (C) When Rho-3 is localized exclusively to the ER by a KDEL tag (GFP–Rho-3–KDEL) it fails to rescue the *rho-3* mutant phenotype. (D) An ER-retained form of Rho-1 (GFP–Rho-1–KDEL) fails to rescue the *rho-3* mutant phenotype. (E and F) Spi does not require cleavage for translocating in the axons. Spi–GFP was expressed in the eye disc by GMR–Gal4, and its distribution in axons in the optic stalk (o.s.) was monitored. (E) Wild-type Spi–GFP expressed in a wild-type genetic background is detected throughout the axons. (F) Mutating the Rhomboid cleavage site in Spi–GFP does not alter its distribution in axons. (G) Cleavage of Spi in the ER does not occur in *rho-3* mutants, yet the distribution of the ligand in axons is similar to wild-type. (H) Quantification of Spi distribution in axons. Mean pixel intensities were determined at the entry point of the optic stalk into the brain, and at the eye disc. A ratio of mean pixel intensity in the eye to mean pixel intensity in the optic stalk was calculated per specimen; 7–10 specimens were used for each quantification. The differences observed are not significant (ANOVA). Scale bars: 10 µm.

The combined ER and secretory compartment localization of Rho-3 is critical for its ability to promote Spi secretion from axons. We next asked whether the ER component of this localization is sufficient for Rho-3 function in lamina induction. We uncoupled the two localizations by tagging Rho-3 with a KDEL sequence at its luminal C-terminus, thereby retaining it in the ER. This construct, as well as a KDEL-tagged Rho-1, were fused at their N-termini to GFP, and inserted into the same genomic landing site as the constructs previously described. Although GFP–Rho-3–KDEL and GFP–Rho-1–KDEL localize to the ER, and efficiently cleave Spi in cell culture assays and in vivo (unpublished data), they could not rescue the *rho-3* lamina phenotype upon expression in the eye by MT14–Gal4 (Figure 4A–4D). This indicates that the ER localization of Rho-3 is not sufficient to promote EGFR signaling in the lamina, and suggests that the active Spi molecules secreted from the axons are not processed in the ER.

Since Spi that is secreted by photoreceptor axons is not cleaved in the ER, we monitored the capacity to traffic the Spi precursor to axonal termini. GMR–Gal4-driven expression in a wild-type eye disc of the Spi precursor marked with GFP at the N terminus, gave rise to translocation of the GFP tag across the entire length of the axon bundle (Figure 4E and 4H). However, it is not possible to determine by this assay whether the ligand that reaches the axon termini represents the precursor form or the cleaved ligand. Two lines of evidence suggest that the ligand precursor can be trafficked from the cell body to the axon terminus. First, a non-cleavable form of Spi also reached the axonal growth cones, when expressed in the eye disc (Figure 4F and 4H). Second, expression of mSpi–GFP in a *rho-3* mutant background, in which the precursor does not undergo cleavage in the ER, gave rise to a ligand distribution in axons that was similar to wild-type (Figure 4G and 4H). Taken together, these experiments demonstrate that the Spi precursor can be trafficked along the axon, and suggest that it is cleaved outside of the ER prior to its secretion.

To support this conclusion, we assayed the ability of a cleaved form of the ligand (cSpi), which is localized to the ER [17], to rescue the *rho-3* phenotype upon expression by MT14–Gal4 in R2, R5, and R8. Biologically active cSpi, tagged with HA or HRP, failed to induce ElaV expression in *rho-3* laminae (Figure S3). This is consistent with the notion that cleavage of Spi in the ER is not the mode by which Rho-3 promotes secretion, and suggests that the importance of the ER to Rho-3 function stems from a different mechanism.

### The ER Facilitates Rho-3 Trafficking to Axons

The above experiments demonstrate that while ER localization is crucial for the ability of Rho-3 to promote axonal secretion of Spi, the functional ligand is not cleaved in the ER. We therefore examined whether the ER could promote Rho-3-dependent signaling by facilitating the trafficking of the ligand-processing machinery to axons.

**Figure 5:**
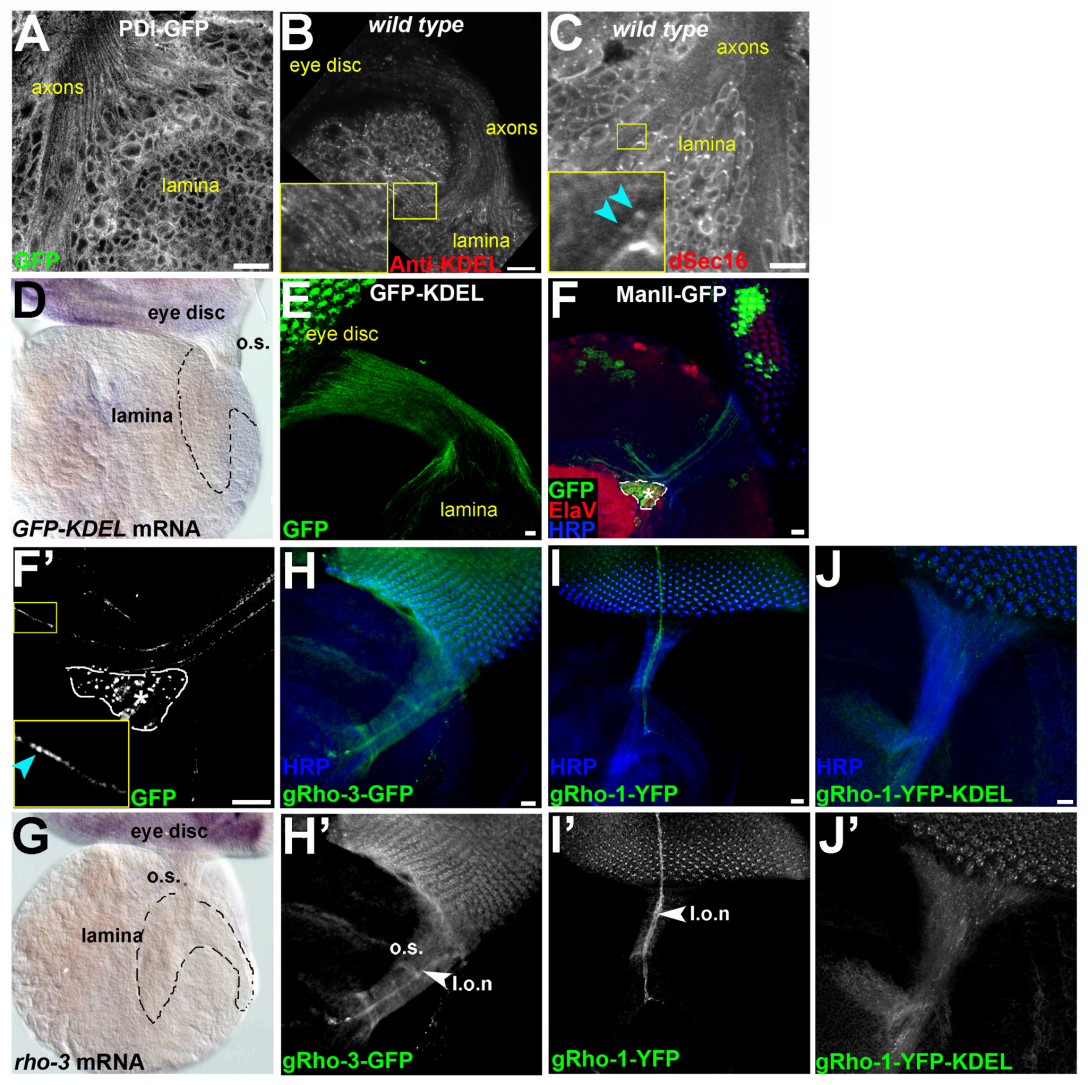
The ER facilitates Rho-3 trafficking to axons. (A–C) Endogenous ER markers are detected throughout the axons of photoreceptor neurons. (A) A GFP gene trap in the endogenous PDI. GFP immunoreactivity is detected along the axons (not shown) and at their termini, as they invade the lamina. (B) ER-retained proteins are revealed by anti-KDEL immunostaining along the length of the axon. The inset shows a magnification of the axonal termini in the lamina. (C) ER exit sites, marked by dSec16, showing a smooth staining and some brighter puncta in the axons and in lamina cells. The inset shows dSec16 puncta (arrowheads) in axons which have reached the lamina. (D and E) Expression of GFP–KDEL in the eye disc by mδ0.5–Gal4. (D) RNA in situ hybridization with a *GFP* probe, showing that *GFP–KDEL* mRNA is restricted to cell bodies in the eye disc. No signal is detected in the optic stalk (o.s.) or the lamina (outlined). (E) GFP–KDEL protein can reach the axon through the ER, and is detected along the entire length of the axon. (F) ManII–GFP (green), expressed in wild-type MARCM clones, is present throughout the axon. The outlined area (asterisk) is a clone in the lamina cells. (F′) shows the ManII–GFP separately, with an enlargement of one fascicle. The Golgi is detected as discrete units (arrowhead), with a “beads on a string” appearance. (G) *rho-3* mRNA is confined to cell bodies in the eye disc, and is not detected in the axonal projections into the lamina (outlined). (H) The ER localized gRho-3–GFP (green, and in [H′]) is localized to the eye disc, and is also enriched in axons. Arrowhead in (H′) marks the larval optic nerve (l.o.n.) where nonspecific staining occurs. (I) gRho-1–YFP (green, and in [I′]) is localized specifically to the eye disc, and does not reach the axons. Arrowhead in (I′) marks the larval optic nerve. (J) When gRho-1–YFP is targeted to the ER (gRho-1–YFP–KDEL, green and in [J′]), it is translocated along the axon bundle. Scale bars: 10 µm.

Examination of the endogenous ER markers protein disulfide isomerase (PDI) and BiP reveals that the ER extends throughout the axons of developing photoreceptor neurons (Figure 5A and unpublished data), as does the detection of KDEL-tagged ER luminal proteins (Figure 5B). ER markers were also observed in axons of adult flies (unpublished data), consistent with previous reports indicating that the ER is continuous in the axons of various neurons [26],[42]. We also detected the presence of endogenous ER exit sites (marked by dSec16 [43]) along the axons and at their termini in the lamina (Figure 5C), suggesting that proteins are released from the ER in these locations. Consistently, Golgi outposts (marked by Mannosidase II (ManII)–GFP [44]) were also evident along the entire axon length (Figure 5F). These observations suggest that in photoreceptor axons, the ER can be used by secreted proteins to reach a given exit site, prior to progressing along the secretory pathway.

To further test this idea, we expressed an ER-localized GFP (GFP–KDEL) [45] in the eye disc. GFP immunofluorescence was observed throughout the axons, while *GFP* mRNA was confined to the cell bodies (Figure 5D and 5E). Thus, proteins localized to the ER in the cell body can also reach the axon, by utilizing the extension of the ER to the axon.

Since Rho-3 is ER localized in the cell body, it could use this compartment in a manner similar to GFP–KDEL to move distally. Indeed, whereas *rho-3* mRNA is not detected in the axons (Figure 5G), gRho-3–GFP is found in a continuous distribution in axons (Figure 5H). Conversely, gRho-1–YFP, which is not localized to the peri-nuclear ER, fails to reach the optic stalk (Figure 5I). To examine the possibility that ER localization would promote the axonal delivery of a Rhomboid protease, we generated another gRho-1–YFP construct, with a C-terminal KDEL tag. In contrast to gRho-1–YFP, gRho-1–YFP–KDEL was robustly distributed along the entire length of the axons (Figure 5J).

Taken together, these results imply that the importance of the ER for Spi signaling in this physiological context stems from its ability to promote trafficking to the axons, where Spi processing subsequently occurs.

### Co-Trafficking of Spi, S, and Rho-3 Sensitizes EGFR Signaling in the Lamina to S Levels

**Figure 6:**
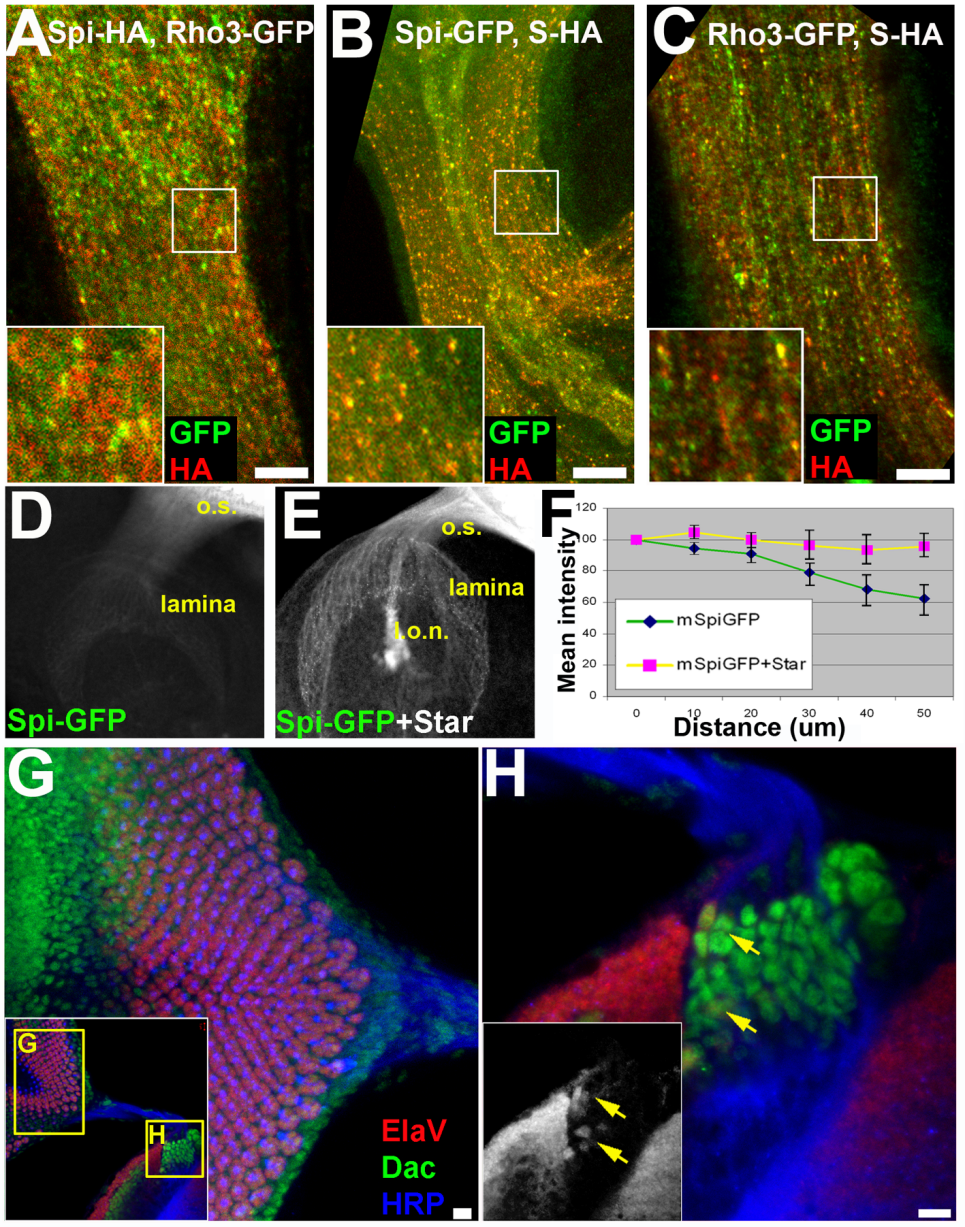
Co-trafficking of Spi, S, and Rho-3 sensitizes EGFR activation in the lamina to *S* gene dosage. (A–C) The localization of Spi, S, and Rho-3 was examined in the optic stalks of specimens expressing HA- (red) or GFP-tagged (green) versions of the proteins in the eye disc by GMR–Gal4. (A) Spi–HA co-localizes with Rho-3–GFP in the axons. (B) Spi–GFP co-localizes with S–HA. (C) S–HA co-localizes with Rho-3–GFP. (D–F) S stabilizes Spi during their joint axonal trafficking. (D) The levels of Spi–GFP (green), expressed on its own in the eye disc, decay along the axons. (E) Co-expression of S–HA with Spi–GFP stabilizes the ligand. l.o.n., larval optic nerve; o.s., optic stalk. (F) Quantification of the effect of S expression on Spi. Mean pixel intensities of Spi–GFP were determined every 10 µm along the optic stalk, from the point where the optic stalk leaves the eye disc (distance  = 0). GFP intensity was normalized to 100 at point 0. Seven specimens were examined per genotype. Student′s *t*-test shows that the difference at the most distal point is statistically significant. (G and H) EGFR signaling is more sensitive to S levels in the lamina than in the eye. EGFR activation in both tissues is assayed by ElaV expression (red), Dac (green), and HRP (blue). (G) *S* heterozygous eye disc. EGFR phenotypes associated with *S^+/−^* (misrotated ommatidia and missing photoreceptors) lead to the slightly abnormal appearance of ElaV staining, but the phenotype is not severe. The inset shows that photoreceptor axons extend normally to the brain. (H) The lamina of the same specimen as in (G) shows a severe reduction in EGFR activation. Only a small number of cells (arrows) at the posterior of the lamina express ElaV, although Dac expression is unperturbed. Scale bars: 5 µm.

Besides Rho-3, Spi and S are also localized to the ER in the eye disc. Therefore, the three components could associate in this compartment for joint trafficking to the axons. To test this hypothesis, we examined the co-localization of biologically active, HA-tagged versions of Spi or S with Rho-3–GFP. S–HA co-localizes with Spi–GFP in the axons at the optic stalk (Figure 6B). In photoreceptor cell bodies S was shown to stabilize Spi [46].We observed that S stabilizes Spi in axons, and promotes its trafficking through the axons, as more Spi–GFP molecules arrive at the lamina when co-expressed with S–HA (Figure 6D–6F). S–HA also co-localizes with Rho-3–GFP in the axons. Both the ligand and chaperone thus appear to co-localize with Rho-3–GFP in axons traveling through the optic stalk (Figure 6A and 6C).

We have previously shown that S is a substrate for ER-localized Rhomboid proteases [23], and that cleaved S cannot traffic Spi [20]. ER-based cleavage of S has a functional significance, as it limits the trafficking of the Spi precursor by the S chaperone out of the ER. This results in an increased sensitivity of EGFR signaling to S levels. Indeed, *S* heterozygous flies exhibit reduced EGFR signaling during oogenesis and eye development, where the ER-active Rho-2 and Rho-3 mediate Spi processing, respectively [23]. Thus, a sensitivity to *S* levels is indicative of exposure to Rhomboid-based cleavage in the ER. We find that *S* heterozygous flies show a severe reduction in ElaV expression in the lamina (Figure 6G and 6H). Importantly, the defect in EGFR signaling in the laminae of these flies is significantly more severe than the compromised induction of photoreceptors in the eye disc. This may reflect a longer exposure of S to ER cleavage by Rho-3 during trafficking to the axon termini. Thus, the hypersensitivity of the lamina to *S* gene dosage supports the notion that S and Rho-3 are jointly trafficked through the ER in photoreceptor axons.

### Endosomal Trafficking Regulates Spi Secretion

**Figure 7:**
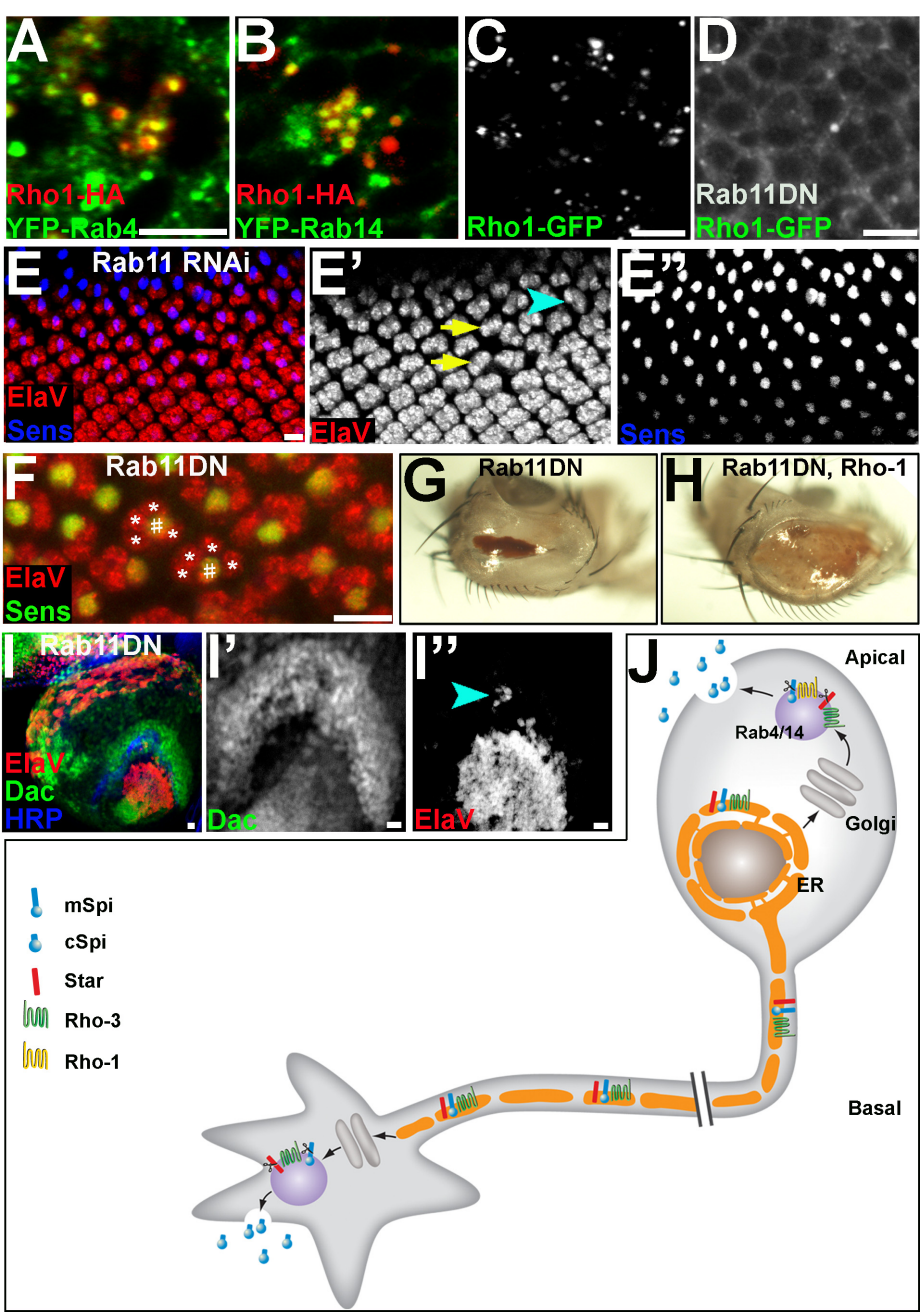
Regulation of Spi secretion by endosomal trafficking. (A and B) The apical Rho-1 puncta are Rab4/14 endosomes. Rho-1–HA (red), expressed in the eye disc, co-localizes with YFP-tagged Rab4 or Rab14 (green). (A) Co-localization of Rho-1–HA and YFP–Rab4. (B) Co-localization of Rho-1–HA and YFP–Rab14. (C) Rho-1–GFP shows the typical punctate staining of Rab4/14 endosomes when expressed in the eye disc. (D) Upon co-expression of Rab11DN, Rho-1–GFP is mislocalized. Some weak punctate staining is still detectable, but most GFP immunolabeling appears sub-membranal. (E) Expression of Rab11 RNAi in the eye disc by GMR–Gal4 yields EGFR phenotypes such as missing photoreceptors (arrows in [E′]), misrotated ommatidia (arrowhead), and defective ommatidial spacing. Importantly, although ElaV staining (red, and in [E′]) reveals these defects, R8 differentiation (Senseless, blue, and in [E″]) is unaffected. (F) R8-specific expression of Rab11DN by Sca–Gal4 disrupts EGFR signaling in adjacent cells, indicating that Rab11 is involved in ligand secretion. For example, two ommatidia with only four outer photoreceptors (*) and an R8 cell (#) are marked. (G) Adult flies expressing Rab11DN by GMR–Gal4 have small eyes. (H) Co-expression of Rho-1 or Rho-3 (not shown) rescues the defects associated with Rab11DN, suggesting that these defects are partly due to a failure to properly target Rhomboids and secrete Spi. (I) Expression of Rab11DN in the eye also leads to a defect in EGFR signaling in the lamina. ElaV staining in the lamina (red, and in [I″]) is strongly reduced (arrowhead in [I″] shows residual staining), while Dac expression (green, and in [I′]) is not affected. (J) Model. ER-facilitated trafficking of the Spi-processing machinery to axon termini promotes Spi secretion from the axons to the lamina. Scale bars: 5 µm.

Following its trafficking to the axonal termini, Spi seems to be secreted locally at a precise location [6]. In the eye disc, Spi is also secreted locally, from a late secretory compartment where Rho-1 and Rho-3 reside [23]. To gain insight into the mechanism of Spi release, we set out to identify the “late compartment” in the eye disc. A variety of compartment markers were tested for co-localization with Rho-1–HA expressed in the eye disc (see also [23]), including a collection of YFP-tagged Rab proteins [47]. The only significant co-localization was observed with YFP–Rab4 and YFP–Rab14 (Figure 7A and 7B). This co-localization was also verified in cell culture, where a significant proportion of Rho-1-, Rab4-, and Rab14-positive puncta overlap (Figure S4A). YFP–Rab4 and YFP–Rab14 also co-localize with apical, but not peri-nuclear, Rho-3–HA staining in the eye disc (Figure S5).

Interruption of Rab4 and Rab14 function in photoreceptors by RNA interference (RNAi) or dominant negative (DN) approaches did not result in any discernible phenotypes. However, both Rab proteins interact with effectors of Rab11 [48],[49], suggesting a role for this major conserved regulator of endosomal trafficking in Spi exocytosis. Indeed, expression of a DN form of Rab11 in *Drosophila* cell culture disrupted the morphology of Rab4/14 endosomes, marked by Rho-1–red fluorescent protein (RFP) or Spi–HA, when the latter was co-expressed with S (Figure S4). Furthermore, in the eye imaginal disc, Rho-1–GFP, which is normally localized to discrete puncta, is misocalized upon co-expression of Rab11DN by GMR–Gal4 (Figure 7C and 7D). Thus, although Rab11 does not co-localize to the Rho-1-containing endosomes, its function is essential for their correct formation.

We then asked whether EGFR signaling is affected by impairment of the Rab4/14 compartment. Indeed, expression of Rab11DN by GMR–Gal4 led to a reduction in the number of ElaV-expressing cells in the eye disc (unpublished data), as did expression of a Rab11 RNAi construct (Figure 7E). Importantly, there was no alteration of photoreceptor R8 differentiation, which is not dependent upon EGFR signaling. Since this phenotype may reflect a requirement for Rab11 in the signal-receiving cells, downstream to EGFR, we expressed the Rab11DN construct specifically in R8, which is the only photoreceptor that acts exclusively as a signal-emitting cell. Again, EGFR phenotypes such as missing photoreceptors and mis-rotated ommatidia were readily apparent (Figures 7F, S6A, and S6B). This indicates that Rab11 acts non-autonomously in R8, where it is required for EGFR ligand secretion.

When larvae expressing UAS–Rab11DN by GMR–Gal4 in the eye disc were allowed to develop, the resulting adults had very small and rough eyes, as previously reported (Figure 7G; see also [47]). Although Rab11 has pleiotropic functions, this phenotype is at least partly due to a specific failure in EGFR ligand secretion, since co-expression of Rho-1 with Rab11DN considerably ameliorated the phenotype (Figure 7H). We conclude that in the eye disc, Spi is cleaved and secreted from Rab4/14 endosomes, and that the normal function of these endosomes is required for EGFR ligand trafficking and processing.

The requirement for Spi cleavage to take place after ligand is trafficked out of the ER in both the cell bodies and axons, raised the possibility that subsequent trafficking steps also share common features. We therefore sought to determine whether Spi secretion from the axons similarly involves Rab4/14 endosomes, and is dependent upon Rab11 function. Indeed, we found that Rab4 or Rab14, expressed in the eye disc by GMR–Gal4, reached axonal growth cones, as did Rab11. Note that GMR–Gal4 does not drive expression in the lamina ([6] and Figure 4E–4G). As in the eye disc, co-localization between Rho-3–HA and YFP–Rab4 or YFP–Rab14 was observed in axonal termini (Figure S5), but not along the length of the axons in the optic stalk (unpublished data).

Expression of Rab11DN in the eye disc by GMR–Gal4 led to a significant reduction in the number of ElaV-positive cells in the lamina, while Dac expression was normal (Figure 7I). Importantly, expression of Rab11DN in R8, which does not secrete Spi to the lamina, severely impairs EGFR signaling in the eye disc but not in the lamina (Figure S6). To further separate the axonal function of Rab11 from its requirement in photoreceptor differentiation, we expressed Rab11DN by GMR–Gal4 together with RasV12, which induces massive photoreceptor recruitment ([50] and Figure S7A). In the eye disc RasV12 was epistatic to Rab11DN, where all cells were converted to ElaV-expressing neurons, supporting the notion that Rab11 acts upstream to Ras (Figure S7). Expression of RasV12 in the eye induces an enlarged lamina with extra lamina neurons. Co-expression of Rab11DN attenuated the effects of RasV12 on lamina development in seven of 12 specimens, leading to wild-type or even reduced ElaV expression (Figure S7). In other words, we have uncoupled the requirement for Rab11 for secretion of the ligand in the eye disc and in the lamina by using RasV12 to bypass the requirement for the ligand in the eye disc. Therefore, this effect specifically represents the requirement for Rab11 to allow secretion of the ligand at the axon termini. This is consistent with the notion that after trafficking of mSpi, S, and Rho-3 to the axonal termini, secretion occurs in a similar manner to the eye disc, utilizing a Rab11-dependent mechanism.

## Discussion

### Axonal Release of Spi Requires the ER Residence of Rho-3

Polarized secretion of ligands from a signal-emitting cell to the appropriate receptive field is crucial for correct intercellular communication. Control over EGFR ligand secretion, and consequently EGFR activation, in *Drosophila* is achieved through trafficking and compartmentalization of the ligand-processing machinery. This work identifies a link between the subcellular localization of the Spi-processing machinery and the polarized release of Spi from axons.

Subcellular localization of Rhomboid proteases, which process the inactive Spi precursor, impinges on ligand secretion [23]. Both Rho-1 and Rho-3 are localized to apical Rab4/14 endosomes, where they are redundant in promoting Spi release from cell bodies. In contrast, only the Rho-3 protease mediates axonal secretion of Spi. This is evident from the *rho-3* mutant phenotype, which shows a complete loss of EGFR activation in the lamina. Since the two proteases are expressed in the neurons which secrete Spi, and share the same substrate specificity, these features cannot account for the specific requirement for *rho-3*. RNA transport and localized translation of Rho-3 are also inconsistent with the following observations: (a) no *rho-3* RNA was detected in axons, (b) gRho-3–GFP, reflecting endogenous expression, is localized throughout the axon, rather than concentrated at a point of localized translation, and (c) Rho-3 cDNA, devoid of 3′ or 5′ UTRs, rescued the mutant phenotype. The RNA of the rescuing construct was also not localized to axons.

Our results indicate that the exclusive requirement for Rho-3 is due to its ER localization. Re-localization of some of the Rho-1 pool to the ER, or removal of Rho-3 from the ER, achieved by swapping specific sequences, alternated their potencies to promote axonal secretion of Spi. Furthermore, when the ER export of endogenous Rho-1 was compromised, EGFR activation was partially restored to the lamina of *rho-3* mutants. Thus, the ER localization of Rho-3 in photoreceptor neurons serves a dual function: it negatively regulates Spi secretion from cell bodies, via premature cleavage of S [23], and positively promotes Spi secretion from the axons to the lamina, by facilitating trafficking of the ligand-processing machinery to axon temini (schematized in Figure 7J).

### The ER Promotes Trafficking of the Spi-Processing Machinery to Axons

How does the ER localization of Rho-3 contribute to Spi secretion from axons? The inability of GFP–Rho-3–KDEL or cSpi–HA to rescue the *rho-3* phenotype demonstrates that the axonally secreted Spi is not cleaved in the ER, and prompted investigation into the role of the ER in promoting axonal trafficking.

We have shown that in *Drosophila* photoreceptor neurons, the ER extends throughout the axons. ER exit sites and Golgi outpost markers were also detected in axons. The continuity of the ER was previously demonstrated in Purkinje neurons [26] and in other cell types, including *Drosophila* oocytes [42],[51]. This implies that ER-localized proteins could use this compartment to move distally in the axon. Indeed, GFP–KDEL expressed in the eye disc reaches the axonal termini. Furthermore, the ER-localized Rho-3 is enriched in axons, as opposed to Rho-1, which is restricted to endosomes. Importantly, restricting the gRho-1 construct to the ER with a KDEL sequence gave rise to a robust translocation of the protease throughout axons, reaching their growth cones in the lamina.

ER-facilitated trafficking of Rho-3 could occur through diffusion in the ER membrane, with exit and retrieval of ER-derived vesicles being biased distally. Alternatively, and perhaps more likely, the ER presence of Rho-3 could lead it to an exit site localized at the axon base, from which trafficking would be directed towards the growth cones. This would explain the ability of Rho-1 to rescue the *rho-3* phenotype under strong overexpression conditions. Distinction between these possibilities would require co-localization of Rho-3 or Spi immunoreactivity with known compartment markers in axons. So far, and despite a large number of markers examined, we could not detect such co-localization (unpublished data). Since the extension of the ER is correlated with the growth of the axons [52],[53], ER-facilitated trafficking also provides a means of ensuring that ligand is released only once the axons have reached their target layer, and ER exit sites and Golgi membranes are set in place.

Spi, S, and Rho-3 are all localized to the peri-nuclear ER in the eye disc. Since all three proteins can interact with one another [19],[46], this implies that the processing machinery could assemble in the ER for joint trafficking. Indeed, we found that Spi, S, and Rho-3 also co-localize in photoreceptor axons. Further evidence for the joint trafficking of S and Rho-3 is the marked sensitivity of EGFR signaling in the lamina to S levels. We have previously shown that S cleavage in the ER leads to compromised EGFR activation phenotypes upon halving *S* gene dosage [23]. The observation that EGFR signaling in the lamina is even more sensitive to *S* gene dosage than in the eye suggests that Rho-3 and S spend a significant time in the ER, where the chaperone is exposed to inactivation by cleavage.

How targeting of Spi–S–Rho-3 complexes to the basally located axons or the apical Rab4/14 endosomes is achieved is unclear. In the case of Hh, the presence or absence of the C-terminal cleavage fragment in the Hh-containing vesicle determines its destination [4]. The Spi C-terminus is not required for axonal targeting, since a Spi–GFP construct lacking most of the C-terminus showed the same distribution as intact Spi–GFP upon expression in the eye (unpublished data). Alternatively, another factor, which would be ER localized, could promote the trafficking of the processing machinery to axons. This factor is also expected to be expressed mainly in R2, R5, and R8, accounting for their importance in Spi secretion to the lamina. In the *Drosophila* oocyte, the polarized ER exit of another EGFR ligand, Gurken, is regulated by Cornichon. Somatic functions for Cornichon and its homolog Cornichon related have also been identified but not thoroughly explored yet [54].

While the presence of ER markers in axons or dendrites has been previously reported [27], the biological significance of such observations, commonly derived from protein localization data in cultured neurons, could only be speculated upon, since no functional readout was examined. The unique properties of photoreceptor axons in *Drosophila*, which not only conduct electrical signals but are also involved in transmitting developmental cues at an earlier phase, have allowed us to functionally demonstrate the essential role of the ER in trafficking the complete EGFR ligand-processing apparatus to axon termini. This mechanism is clearly distinct from the established roles of the axonal ER in allowing local translation of secreted or transmembrane proteins whose mRNAs are enriched at axon termini.

### Endosomal Regulation of Spi Secretion

Spi is released to the extracellular milieu following cleavage by Rho-1. Different experimental systems have yielded conflicting reports as to the compartment in which the protease resides [18]–[20],[55]. We now find that in both photoreceptor neurons and Schneider cells, Rho-1 is localized to an endosomal population marked by Rab4 and Rab14. Rab4 localizes to fast recycling endosomes, which mediate the retrieval of endocytosed cargo to the plasma membrane [56],[57]. Rab14 mediates trafficking between the Golgi and endosomes [58],[59]. Both Rab4 and Rab14 share binding proteins with Rab11 [48],[49], a major regulator of vesicle transport.

The role of endosomal dynamics in Spi secretion is manifested by the EGFR phenotypes obtained following expression of Rab11 RNAi or DN constructs. While Rab11 has pleiotropic functions and is not dedicated to EGFR signaling, perturbing Rab11 directly impinges on Spi secretion. This was evident from the mislocalization of Rho-1–GFP in Rab11DN-expressing photoreceptors, and from similar effects in cell culture. This mislocalization is likely the cause of the phenotype, since co-expression of Rho-1 or Rho-3 with Rab11DN abrogated the small eye phenotype associated with Rab11DN expression. Although interfering with endosomal dynamics may also perturb signaling downstream of the receptor, we did not observe a mislocalization of EGFR itself (unpublished data). Furthermore, the expression of Rab11DN in R8 impaired the differentiation of nearby cells into photoreceptor neurons, demonstrating that Rab11 acts non-autonomously upstream of the receptor, consistent with a role in ligand secretion.

Rho-1 and some of the Rho-3 pool are localized to Rab4/14 endosomes. The intracellular route by which they reach these compartments remains to be explored. From the ER accumulation of Rho-1–HA in *sed5* mutant clones, we infer that the proteases do not undertake a Golgi-independent route to the Rab4/14 endosomes [41]. Furthermore, Rab14 mediates trafficking between the Golgi and endosomes [59], and Rab11 endosomes can be reached without passing through the plasma membrane (see for example [60]–[62]). Therefore, there is no indication that Rhomboids must pass through the plasma membrane to reach the endosomal compartment. Nevertheless, if Spi is secreted by fusion of Rhomboid-containing endosomes with the membrane, then retrieval by endocytosis should play a role in shaping the steady-state distribution of Rhomboids. Accordingly, we have found that upon expression of a DN form of the Dynamin Shibire, Rho-1–HA immunofluorescence is detected on the plasma membrane (unpublished data).

Trafficking of Spi to endosomes also provides an efficient means of disposing of the ligand in cells that do not express a Rhomboid protease, to prevent nonspecific cleavage on the plasma membrane. In this case, the membrane-bound precursor could be sorted to a membrane domain that segregates to multi-vesicular bodies, and then degraded in the lysosome. Accordingly, distinct membrane domains have been described for Rab4 and Rab11 endosomes [63].

Finally, we detected a co-localization between Rab4/14 and Rho-3 at axonal termini, but not in the optic stalk, and found that disrupting Rab11 function in the eye disc compromised EGFR signaling in the lamina. This effect was not due to defects in eye development, as Rab11DN expressed in R8 also impaired eye development but had no effect on the lamina. This finding raises the possibility that the final steps of secretion from axonal termini and cell bodies are regulated in a similar manner, although Rab11 seems to play a more prominent role in secretion from cell bodies. A precedent supporting such a hypothesis is the requirement for Sec15, which interacts with Rab11, for the localization of several molecules at both photoreceptor cell bodies and axonal termini [64].

In summary, our results describe a mechanism of ER-facilitated trafficking of secreted molecules in axons, prior to processing and secretion at the axon tip. This mechanism could also be utilized for other proteins that are secreted in a polarized manner in neurons.

## Materials and Methods

### DNA Constructs

For the generation of gRho-1–YFP and gRho-3–GFP, 40–45 kb from the *rho-1* or *rho-3* loci, encompassing the ORFs and flanking region, were cloned into P[acman–attB, AmpR] by recombineering-mediated gap repair [35]. The domains extend between 3L:1437674 and 1475379 and 3L:1355719 and 1397235 (release 5.23) for *rho-1* and *rho-3*, respectively. A YFP tag or a YFP–KDEL was inserted at the *rho-1* C-terminus by GalK positive/negative selection [65]. *rho-3* was GFP tagged at the C-terminus using the PL452 C-EGFP tag template vector [66]. Both constructs were injected into VK00005 landing site.

For GFP–Rho-1, GFP–Rho-3, GFP–R1L1-R3, and GFP–R3L1-R1, eGFP was cloned into pUAST–attB at the BglII–EcoRI sites. cDNAs were then cloned using EcoRI and XhoI. All constructs were sequenced, and injected into attP18 lines [39].

cSpiHA contains a triple HA tag from pTWH, inserted after the Spi cleavage site.

mSpi–HA was generated by a site-directed mutagenesis insertion of an XhoI site after T58 of Spi, into which a triple HA tag was subsequently inserted.

mSpi–GFP^mut^ was obtained from S. Urban [33], and cloned into pTWM. Cleavage assays in S2 cells verified that this construct cannot undergo Rhomboid-dependent cleavage (unpublished data).

S–HA is the S cDNA cloned into pTHW. mSpi–GFP and cSpi–HRP were previously described [17],[19]. The cleavage activity of all Rhomboid constructs has been tested in cell culture, and the biological activity of all UAS-based constructs was assayed by expression in wing or eye imaginal discs.

### Immunohistochemistry

Climbing late third-instar larvae were dissected and fixed in PBS containing 4% PFA. All subsequent washes and antibody incubations were done in PBS with 0.1% Triton X-100.

Primary antibodies used were anti-FasIII (mouse, 1∶50), anti-EGFR (rat, 1∶1,000), anti-Senseless (guinea pig, 1∶2,000; from H. Bellen), anti-dSec16 (rabbit, 1∶1,000; from C. Rabouille), anti-Myc (mouse, 1∶100; Santa Cruz Biotechnology), anti-GFP (chick, 1∶2,000; Abcam), anti-HA (mouse, 1∶1,000; Roche), and anti–Troponin H to detect BiP (rat, 1∶100; Babraham Bioscience Technologies). Anti-ElaV (rat, 1∶2,000, or mouse, 1∶500) and anti-Dac (mouse, 1∶500) were obtained from the Developmental Studies Hybridoma Bank, University of Iowa. Cy-5-conjugated goat anti-HRP, as well as Cy-2-, Cy-3-, and Cy-5-conjugated secondary antibodies (1∶200) were obtained from Jackson ImmunoResearch.

In situ hybridizations using *rho-3* or *GFP* probes were done using standard techniques.

### Fly Strains

The following lines were used: GMR–Gal4, Sca–Gal4, m™–Gal4 (from M. Mlodzik), Lz–Gal4, K25–Gal4, MT14–Gal4 ([34], from I. Salecker), UAS–GFP–KDEL [45], MS1096–Gal4, PDI–GFP [67], *sed5^AR113^* (From C. Rabouille), *S^IIN23^*, a collection of YFP-tagged, native or DN UAS–Rab transgenes [47], UAS–Rab11DN (from M. Gonzalez-Gaitan), UAS–ManII–GFP (from Y. Jan), and UAS–Rab11–RNAi (VDRC22198). Null alleles of *rho-1* (*rho-1* ^Δ*p38*^) and *rho-3* (*ru^PLLb^*) were recombined with *FRT2A*, and crossed to *ey–Gal4,UAS–FLP/Cyo;FRT2a,GMR–hid,l(3)CL–L^1^/TM6B* to generate entirely mutant eyes [28]. To generate *sed5^ AR113^* MARCM clones expressing Rho-1–HA, *C155–Gal4,UAS–CD8GFP,hsFLP;Gal80,FRT40A* females were crossed to *sed5^ AR113^,FRT40A/+;UAS–Rho-1HA/+* males. Wild-type clones were generated with a chromosome bearing only *FRT40A*. Clones expressing ManII–GFP were induced in animals of the following genotype: *C155–Gal4,hsFLP/+;UAS–ManII–GFP/+;FRT82B*.

UAS–mSpi–GFP^mut^, UAS–cSpi–HA, UAS–mSpi–HA, UAS–GFP–Rho-1, UAS–GFP–Rho-3, UAS–GFP–R1L1-R3, and UAS–GFP–R3L1-R1 were generated by standard P-element or phi31 germline transformation procedures.

ERG recordings were performed as described in [29].

## Supporting Information

Figure S1 ***rho-3*** **mutants have functional photoreceptors but no post-synaptic responses.** (A) ERG recording from a wild-type fly shows depolarization of photoreceptors in response to light, as well as “on/off transients” (arrowheads), which represent the post-synaptic response of lamina neurons. (B) *rho-3* mutant photoreceptors depolarize in response to light. The lower amplitude of depolarization probably stems from the disorganization of *rho-3* eyes. Importantly, no “on/off transients” can be detected in the mutant (arrowheads), consistent with a failure in lamina neurogenesis. (C) *rho-1* EGUF clones show a wild-type ERG. (D) EGFR endocytic puncta (arrows in D′) are detected in wild-type lamina. (E) *rho-3* mutants have no endocytic EGFR puncta in the lamina. (F) Lamina from *rho-1* EGUF clones show an EGFR distribution identical to wild-type eyes.
(2.92 MB TIF)

Figure S2 ***rho-3*** **RNA is not transported in photoreceptor axons.** (A) RNA in situ hybridization with a *rho-3* probe (see also Figure 5G). *rho-3* RNA is localized to the eye disc and is not detected in axons or in the lamina (outlined). (B) A Rho-3–GFP transgene, expressed in the eye disc under the control of the strong promoter GMR–Gal4 fully rescues the *rho-3* mutant lamina phenotype (arrowhead and outline in inset). Importantly, the transgene contains only the cDNA protein coding sequences, and is devoid of 3′ or 5′ UTRs. Anti-HRP staining (blue) shows axons, Dac (green) marks all lamina cells, and ElaV (red, and shown separately in the inset) marks the lamina cartridge neurons. Scale bar: 10 µm. (C) RNA in situ hybridization with a *GFP* probe on a visual system of the same genotype as in (B). RNA of the rescuing transgene is localized exclusively to the eye, and is not detected in the axons or lamina.
(3.50 MB TIF)

Figure S3 **Cleaved Spi, expressed in the eye disc, does not rescue the** ***rho-3*** **phenotype.** (A–C) Two independent lines of UAS–cSpi–HA (A and B) or a UAS–cSpi–HRP (C) do not rescue the *rho-3* phenotype in the lamina. All constructs were driven by MT14–Gal4, in a *rho-3* mutant background. ElaV is red, Dac is green, and HRP is blue. Scale bars in the upper panels are 20 µm. The lower panels show enlargements of the lamina. Scale bars are 5 µm. Insets in (A) and (B) show anti-HA staining, demonstrating that the constructs are correctly expressed. (D–F) cSpi–HA (D and E) and cSpi–HRP (F) are biologically active, and are potent activators of the EGFR pathway. The activity of the constructs was assayed by their ability to induce extra vein tissue in wings, following induction in the wing pouch by MS1096–Gal4.
(6.10 MB TIF)

Figure S4 **Spi is processed in Rab4/14 endosomes in cell culture.** (A) Rho-1–GFP (green, and in A′), HA–Rab4 (red, and in A′′), and Myc–Rab14 (blue, and in A′′′) co-localize in S2 cells. Scale bar is 10 µm in all panels. (B) Rho-1–RFP (red) marks endosomes in S2 cells. (C) Expression of Rab11DN led to the accumulation of Rho-1–RFP in enlarged, deformed vesicles (arrows). (D) Spi–HA (red) co-expressed with S is used as a marker for the Rho-1 compartment. (E) Upon expression of Rab11DN, Spi–HA is localized to deformed vesicles of the same morphology as in (C).
(1.22 MB TIF)

Figure S5 **Rho-3 co-localizes with Rab4 and Rab14 in cell bodies and growth cones.** (A) Rho-3–HA (red), YFP–Rab4, or YFP–Rab14 (green) co-localize at the apical-most region of photoreceptor cell bodies (upper panels), but not in the peri-nuclear ER (lower panels). Scale bar is 5 µm in all panels. (B) At the growth cones, Rho-3–HA is also co-localized with Rab4/14. No co-localization was observed along the axons at the optic stalk (unpublished data). Note that both Rab4 and Rab14 have a cytoplasmic as well as vesicular distribution. The vesicular distribution overlaps with Rho-3–HA (arrows).
(2.95 MB TIF)

Figure S6 **Rab11 is required non-autonomously in R8 to promote EGFR signaling in the eye but not in the lamina.** (A) Rab11DN expressed in R8 cells by Sca–Gal4. Anti-ElaV staining (red, and shown separately) shows defects in photoreceptor recruitment, ommatidial rotation and spacing—phenotypes associated with compromised EGFR signaling. Importantly, the differentiation of R8 cells, marked with Senseless (green, and shown separately), is not perturbed. HRP (blue) marks axons. Scale bar: 5 µm. (A′) shows an enlargement of the boxed area in (A). (B) Rab11DN expression in R8 does not affect EGFR signaling in the lamina. Despite the defects in eye neurogenesis (A and B), ElaV expression in the lamina is indistinguishable from wild-type. ElaV (red, and shown separately) at the posterior part of the lamina is indicated by an arrowhead. Dac (green) and HRP (blue) mark lamina cell and photoreceptor axons, respectively.
(6.09 MB TIF)

Figure S7 **Rab11 is required for Spi secretion from axons, independently of its function in photoreceptor recruitment.** (A) RasV12 expression in the eye disc induces massive photoreceptor recruitment, and an enlarged lamina with extra lamina cartridge neurons. Anti-ElaV staining (red, and shown separately) decorates photoreceptors in the eye disc and lamina neurons. Dac (green) is expressed in non-neuronal cells in the eye, and in lamina precursors. HRP (blue) marks photoreceptor membranes. Scale bar is 10 µm. (B) Co-expression of RasV12 and Rab11DN. In the eye, RasV12 is epistatic to Rab11DN, indicating that Ras function lies downstream from Rab11. In the lamina, the RasV12 hyperactivation phenotype is suppressed by Rab11DN, suggesting that Rab11DN inhibits lamina neurogenesis independently of its effect on photoreceptor development.
(4.12 MB TIF)

## Abbreviations

Dac: Dachshund
DN: dominant negative
EGFR: epidermal growth factor receptor
EGUF: Eyeless Gal4 UAS Flip
ER: endoplasmic reticulum
ERG: electroretinogram
GFP: green fluorescent protein
gRho-[number]: genomic Rhomboid [number]
Hh: Hedgehog
HRP: Horseradish Peroxidase
ManII: Mannosidase II
PDI: protein disulfide isomerase
RFP: red fluorescent protein
Rho-[number]: Rhomboid [number]
RNAi: RNA interference
S: Star
Spi: Spitz
YFP: yellow fluorescent protein
