# Supplementary figures and images for: Polarized Secretion of Drosophila EGFR Ligand from Photoreceptor Neurons Is Controlled by ER Localization of the Ligand-Processing Machinery
Source: PLoS Biol. 2010 Oct 5;8(10):e1000505. doi: 10.1371/journal.pbio.1000505 (PMC2950126; doi:10.1371/journal.pbio.1000505)

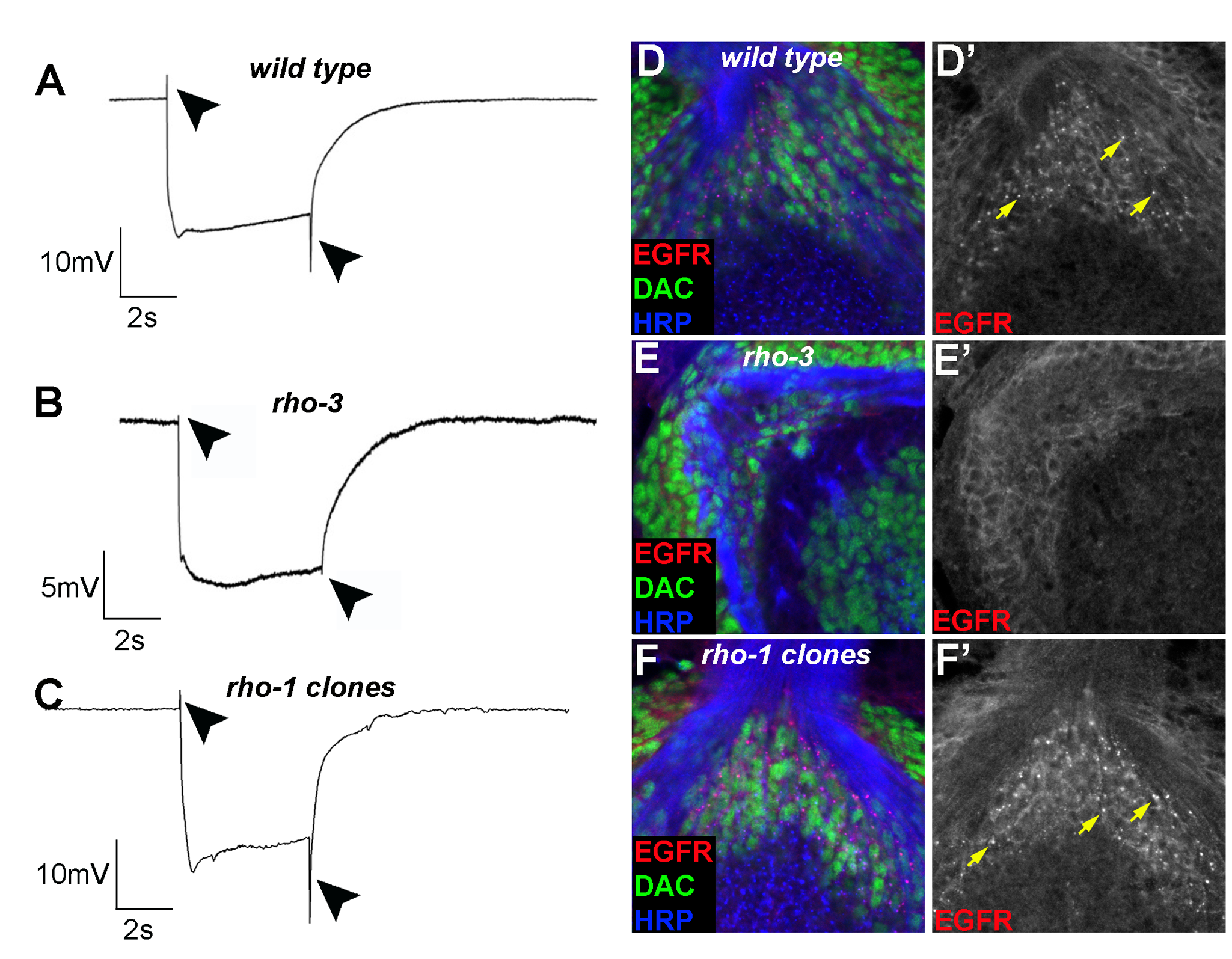

Supplement: Figure S1 — rho-3 mutants have functional photoreceptors but no post-synaptic responses. (A) ERG recording from a wild-type fly shows depolarization of photoreceptors in response to light, as well as “on/off transients” (arrowheads), which represent the post-synaptic response of lamina neurons. (B) rho-3 mutant photoreceptors depolarize in response to light. The lower amplitude of depolarization probably stems from the disorganization of rho-3 eyes. Importantly, no “on/off transients” can be detected in the mutant (arrowheads), consistent with a failure in lamina neurogenesis. (C) rho-1 EGUF clones show a wild-type ERG. (D) EGFR endocytic puncta (arrows in D′) are detected in wild-type lamina. (E) rho-3 mutants have no endocytic EGFR puncta in the lamina. (F) Lamina from rho-1 EGUF clones show an EGFR distribution identical to wild-type eyes. (2.92 MB TIF) [file pbio.1000505.s001.tif]

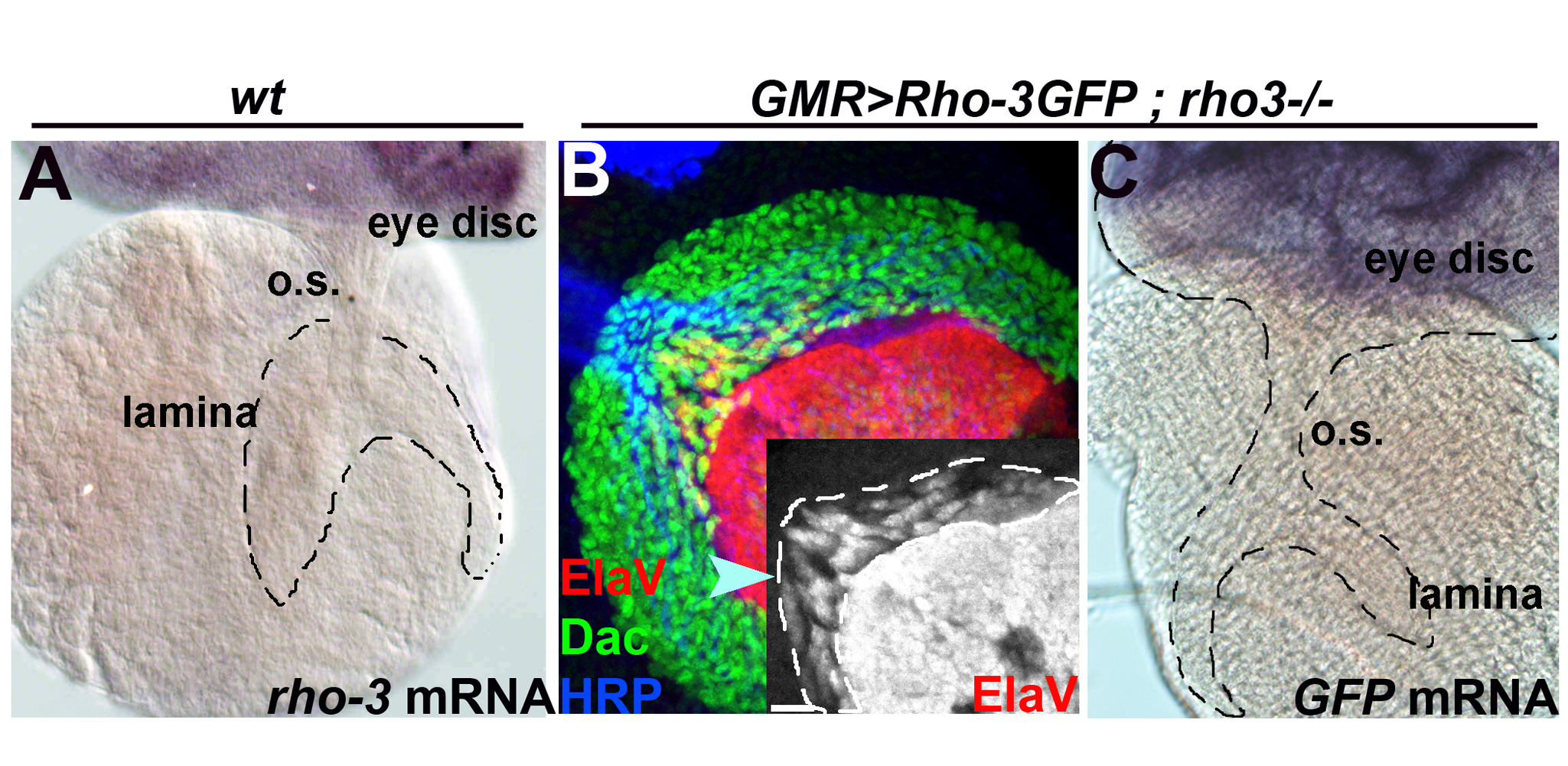

Supplement: Figure S2 — rho-3 RNA is not transported in photoreceptor axons. (A) RNA in situ hybridization with a rho-3 probe (see also Figure 5G). rho-3 RNA is localized to the eye disc and is not detected in axons or in the lamina (outlined). (B) A Rho-3–GFP transgene, expressed in the eye disc under the control of the strong promoter GMR–Gal4 fully rescues the rho-3 mutant lamina phenotype (arrowhead and outline in inset). Importantly, the transgene contains only the cDNA protein coding sequences, and is devoid of 3′ or 5′ UTRs. Anti-HRP staining (blue) shows axons, Dac (green) marks all lamina cells, and ElaV (red, and shown separately in the inset) marks the lamina cartridge neurons. Scale bar: 10 µm. (C) RNA in situ hybridization with a GFP probe on a visual system of the same genotype as in (B). RNA of the rescuing transgene is localized exclusively to the eye, and is not detected in the axons or lamina. (3.50 MB TIF) [file pbio.1000505.s002.tif]

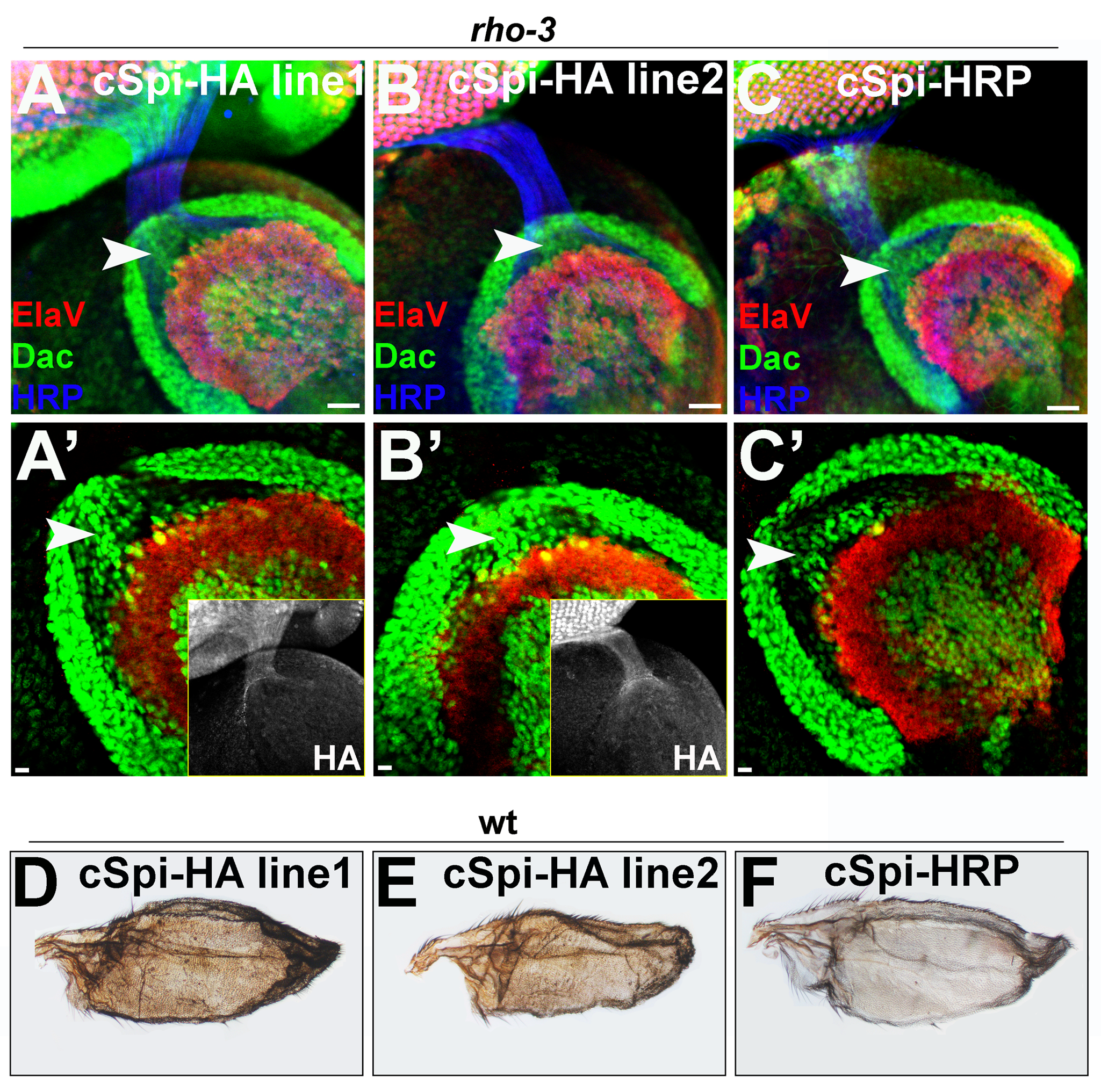

Supplement: Figure S3 — Cleaved Spi, expressed in the eye disc, does not rescue the rho-3 phenotype. (A–C) Two independent lines of UAS–cSpi–HA (A and B) or a UAS–cSpi–HRP (C) do not rescue the rho-3 phenotype in the lamina. All constructs were driven by MT14–Gal4, in a rho-3 mutant background. ElaV is red, Dac is green, and HRP is blue. Scale bars in the upper panels are 20 µm. The lower panels show enlargements of the lamina. Scale bars are 5 µm. Insets in (A) and (B) show anti-HA staining, demonstrating that the constructs are correctly expressed. (D–F) cSpi–HA (D and E) and cSpi–HRP (F) are biologically active, and are potent activators of the EGFR pathway. The activity of the constructs was assayed by their ability to induce extra vein tissue in wings, following induction in the wing pouch by MS1096–Gal4. (6.10 MB TIF) [file pbio.1000505.s003.tif]

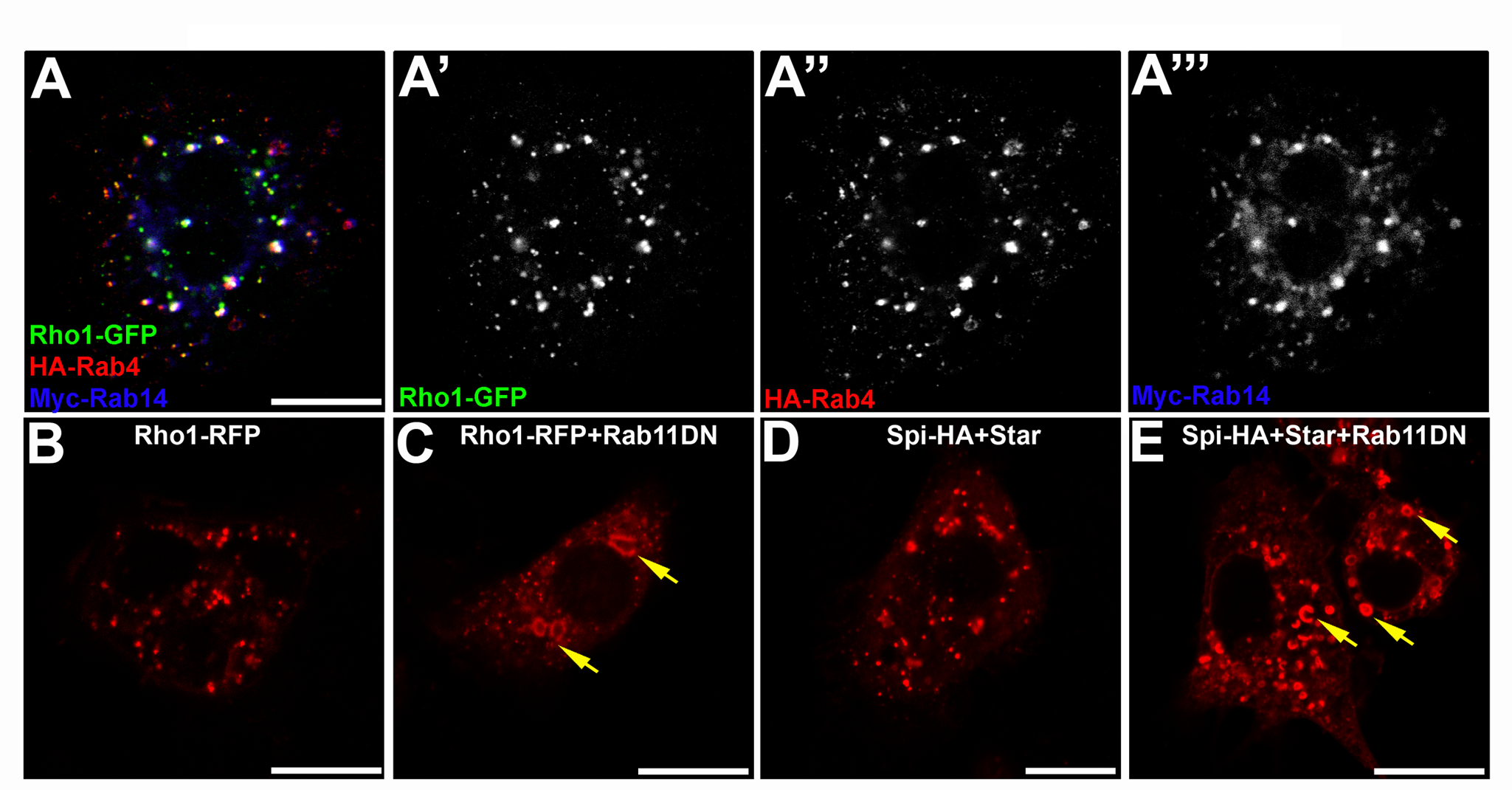

Supplement: Figure S4 — Spi is processed in Rab4/14 endosomes in cell culture. (A) Rho-1–GFP (green, and in A′), HA–Rab4 (red, and in A′′), and Myc–Rab14 (blue, and in A′′′) co-localize in S2 cells. Scale bar is 10 µm in all panels. (B) Rho-1–RFP (red) marks endosomes in S2 cells. (C) Expression of Rab11DN led to the accumulation of Rho-1–RFP in enlarged, deformed vesicles (arrows). (D) Spi–HA (red) co-expressed with S is used as a marker for the Rho-1 compartment. (E) Upon expression of Rab11DN, Spi–HA is localized to deformed vesicles of the same morphology as in (C). (1.22 MB TIF) [file pbio.1000505.s004.tif]

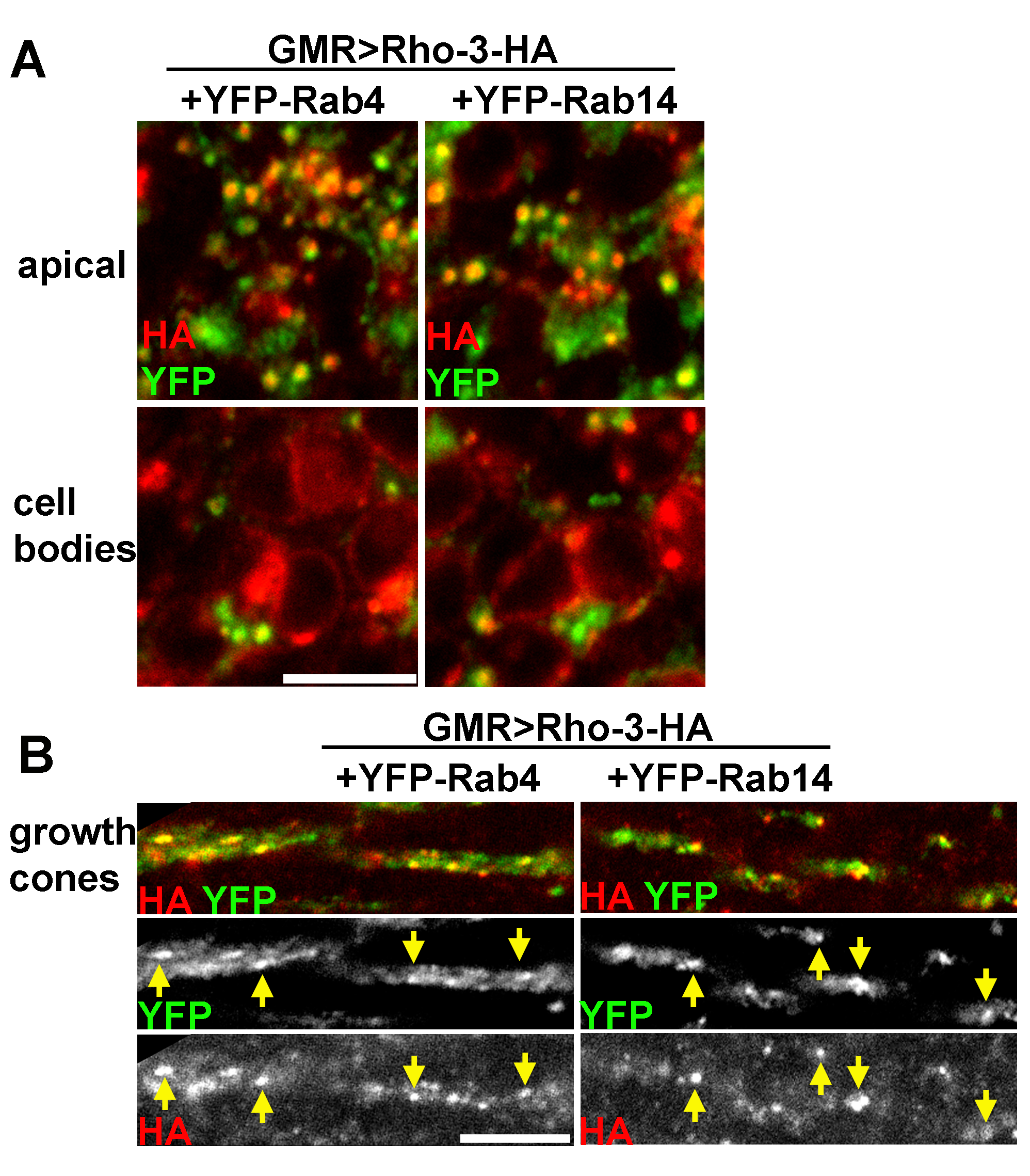

Supplement: Figure S5 — Rho-3 co-localizes with Rab4 and Rab14 in cell bodies and growth cones. (A) Rho-3–HA (red), YFP–Rab4, or YFP–Rab14 (green) co-localize at the apical-most region of photoreceptor cell bodies (upper panels), but not in the peri-nuclear ER (lower panels). Scale bar is 5 µm in all panels. (B) At the growth cones, Rho-3–HA is also co-localized with Rab4/14. No co-localization was observed along the axons at the optic stalk (unpublished data). Note that both Rab4 and Rab14 have a cytoplasmic as well as vesicular distribution. The vesicular distribution overlaps with Rho-3–HA (arrows). (2.95 MB TIF) [file pbio.1000505.s005.tif]

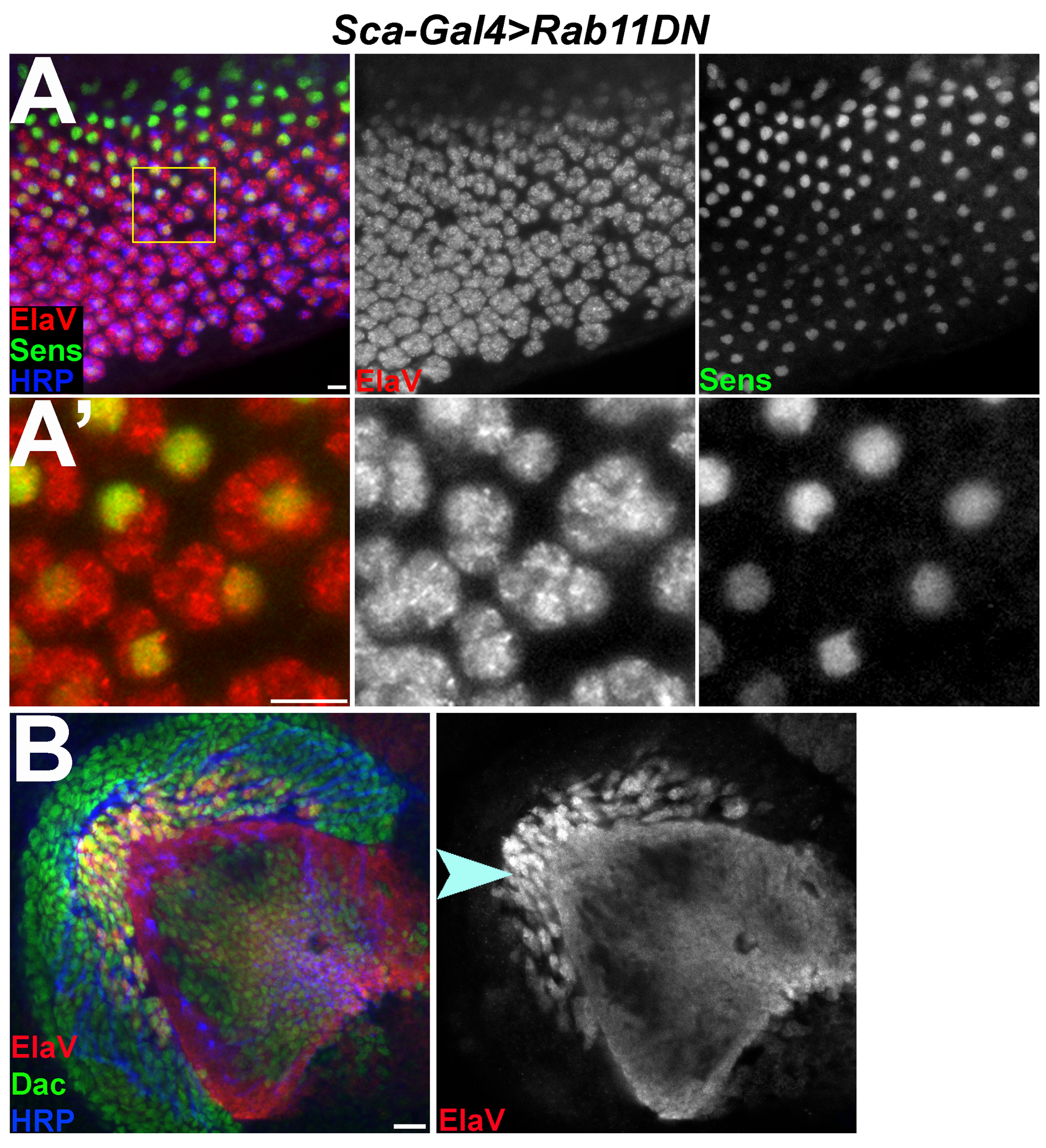

Supplement: Figure S6 — Rab11 is required non-autonomously in R8 to promote EGFR signaling in the eye but not in the lamina. (A) Rab11DN expressed in R8 cells by Sca–Gal4. Anti-ElaV staining (red, and shown separately) shows defects in photoreceptor recruitment, ommatidial rotation and spacing—phenotypes associated with compromised EGFR signaling. Importantly, the differentiation of R8 cells, marked with Senseless (green, and shown separately), is not perturbed. HRP (blue) marks axons. Scale bar: 5 µm. (A′) shows an enlargement of the boxed area in (A). (B) Rab11DN expression in R8 does not affect EGFR signaling in the lamina. Despite the defects in eye neurogenesis (A and B), ElaV expression in the lamina is indistinguishable from wild-type. ElaV (red, and shown separately) at the posterior part of the lamina is indicated by an arrowhead. Dac (green) and HRP (blue) mark lamina cell and photoreceptor axons, respectively. (6.09 MB TIF) [file pbio.1000505.s006.tif]

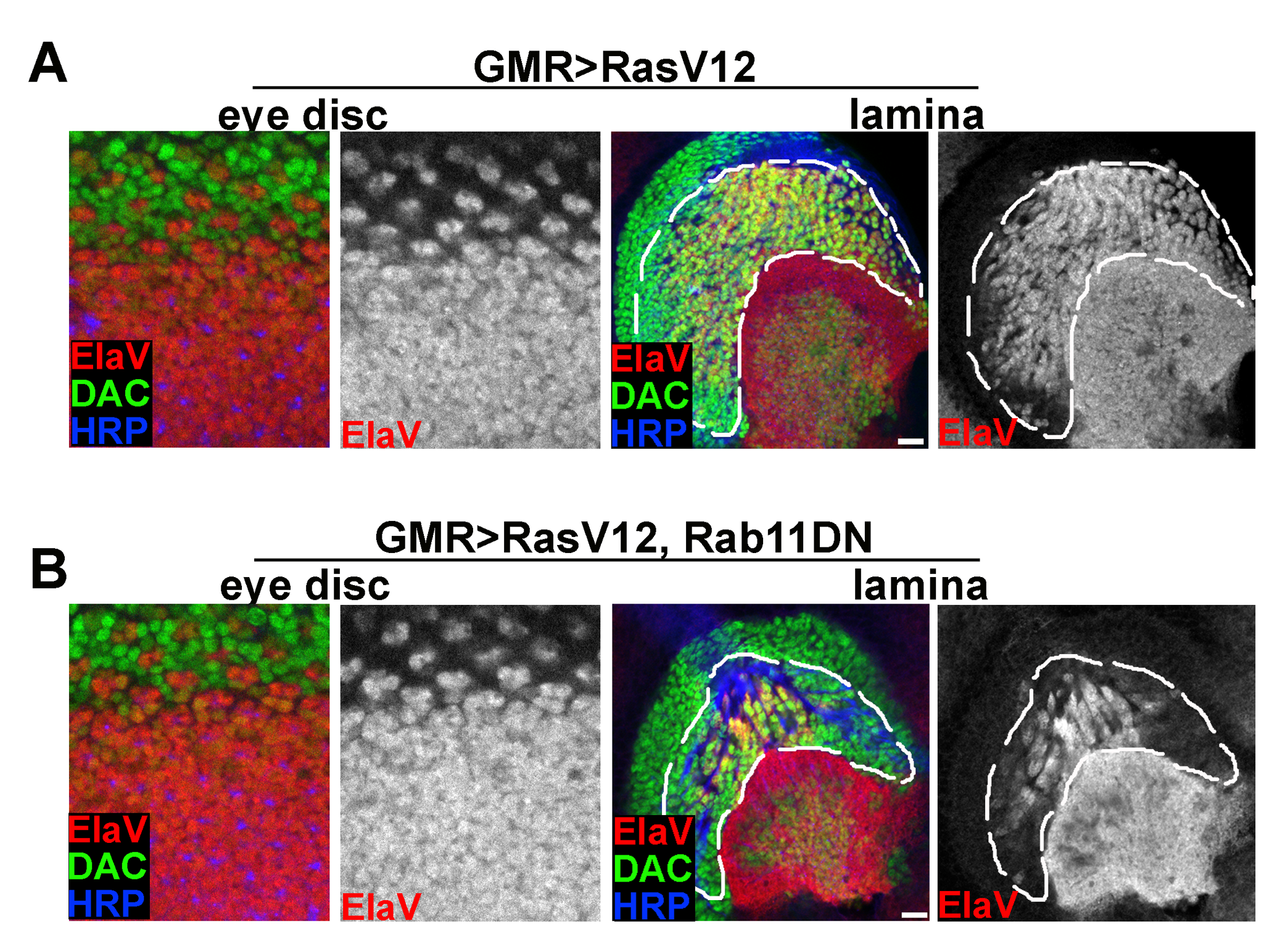

Supplement: Figure S7 — Rab11 is required for Spi secretion from axons, independently of its function in photoreceptor recruitment. (A) RasV12 expression in the eye disc induces massive photoreceptor recruitment, and an enlarged lamina with extra lamina cartridge neurons. Anti-ElaV staining (red, and shown separately) decorates photoreceptors in the eye disc and lamina neurons. Dac (green) is expressed in non-neuronal cells in the eye, and in lamina precursors. HRP (blue) marks photoreceptor membranes. Scale bar is 10 µm. (B) Co-expression of RasV12 and Rab11DN. In the eye, RasV12 is epistatic to Rab11DN, indicating that Ras function lies downstream from Rab11. In the lamina, the RasV12 hyperactivation phenotype is suppressed by Rab11DN, suggesting that Rab11DN inhibits lamina neurogenesis independently of its effect on photoreceptor development. (4.12 MB TIF) [file pbio.1000505.s007.tif]
